# Exploring the Spatial Landscape of the Estrogen Receptor Proximal Proteome With Antibody-Based Proximity Labeling

**DOI:** 10.1016/j.mcpro.2023.100702

**Published:** 2023-12-19

**Authors:** Camilla Rega, Zuzanna Kozik, Lu Yu, Ifigenia Tsitsa, Lesley-Ann Martin, Jyoti Choudhary

**Affiliations:** 1 Division of Breast Cancer Research, The Institute of Cancer Research, London, United Kingdom; 2 Division of Cancer Biology, The Institute of Cancer Research, London, United Kingdom

**Keywords:** proximity labeling, BAR, estrogen receptor, mass spectrometry, protein interactions, breast cancer, endocrine resistance

## Abstract

Estrogen receptor α (ERα) drives the transcription of genes involved in breast cancer (BC) progression, relying on coregulatory protein recruitment for its transcriptional and biological activities. Mutation of ERα as well as aberrant recruitment of its regulatory proteins contribute to tumor adaptation and drug resistance. Therefore, understanding the dynamic changes in ERα protein interaction networks is crucial for elucidating drug resistance mechanisms in BC. Despite progress in studying ERα-associated proteins, capturing subcellular transient interactions remains challenging and, as a result, significant number of important interactions remain undiscovered. In this study, we employed biotinylation by antibody recognition (BAR), an innovative antibody-based proximity labeling (PL) approach, coupled with mass spectrometry to investigate the ERα proximal proteome and its changes associated with resistance to aromatase inhibition, a key therapy used in the treatment of ERα-positive BC. We show that BAR successfully detected most of the known ERα interactors and mainly identified nuclear proteins, using either an epitope tag or endogenous antibody to target ERα. We further describe the ERα proximal proteome rewiring associated with resistance applying BAR to a panel of isogenic cell lines modeling tumor adaptation in the clinic. Interestingly, we find that ERα associates with some of the canonical cofactors in resistant cells and several proximal proteome changes are due to increased expression of ERα. Resistant models also show decreased levels of estrogen-regulated genes. Sensitive and resistant cells harboring a mutation in the ERα (Y537C) revealed a similar proximal proteome. We provide an ERα proximal protein network covering several novel ERα-proximal partners. These include proteins involved in highly dynamic processes such as sumoylation and ubiquitination difficult to detect with traditional protein interaction approaches. Overall, we present BAR as an effective approach to investigate the ERα proximal proteome in a spatial context and demonstrate its application in different experimental conditions.

## Highlights

• BAR enables to investigate the ERα proximal proteome in a spatial context.
• Comparing epitope tag and endogenous antibodies for BAR.
• Interrogating the ERα proximal proteome in endocrine-resistant BC models.
• This study reports a high-confidence ERα proximal protein network.

## Graphical Abstract

**Figure:**
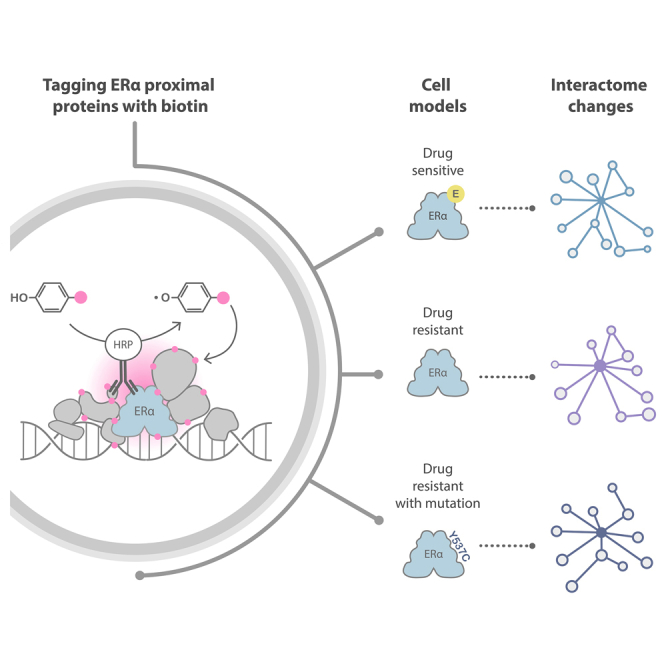

## In Brief

Biotinylation by Antibody Recognition coupled with mass spectrometry is an effective approach to investigate the ERα proximal proteome. The described method successfully detected known ERα interactors and uncovered novel ERα-proximal partners involved in dynamic processes that are often challenging to detect with traditional protein interaction approaches.

Breast cancer affects one in eight women worldwide (1) with over 80% diagnosed with estrogen receptor alpha (ERα) positive breast cancer (2). Once activated by estrogen, ERα translocates to the nucleus and classically associates with estrogen response elements on target genes to control proliferation and cell survival. Transcription activation is regulated through two distinct activation function domains, AF1 and AF2, located in the N-terminal and Ligand Binding Domain (LBD) of ERα, respectively (3). The AF2 activity is dependent on the binding of estrogen, whereas AF1 is modulated by phosphorylation, regulated by various kinases such as MAPK, CDK7, GSK3, and IKKα in response to specific stimuli (4, 5, 6, 7, 8). The diverse genomic functions of ERα are further modulated through the recruitment of several coregulator proteins (9). These include activators, repressors, mediators, chromatin-modifying enzymes, adaptors and other transcription factors that can influence tumor adaptation (10).

The knowledge that estrogen plays a critical role in breast cancer progression has been exploited clinically by the development of targeted therapies designed to disrupt the estrogen signaling axis. These therapeutic strategies either block estrogen synthesis using aromatase inhibitors (11) or antagonize the ERα function. Estrogen antagonists are Selective Estrogen Receptor Modulators (SERMs, such as tamoxifen) which competitively interact with estrogen for binding to ERα (12) as well as Selective Estrogen Receptor Degraders (SERDs, such as fulvestrant) which function by binding to ERα and promote its degradation (13). Although these therapies have significantly improved survival outcomes for patients with ERα positive breast cancer, unfortunately, a considerable number of women display *de novo* resistance to these treatments over time. Nevertheless, in resistant tumors, ERα remains a *bona fide* target by driving ligand-independent transcriptional activation (14), making it a high interest clinical target.

The two main mechanisms of resistance to endocrine therapy are the activation of alternative molecular signals that promote ERα independent cellular proliferation and mutations (15), often located in the LBD (Y537 and D538) of the ERα (16, 17). An additional mechanism of endocrine resistance involves altered interactions of ERα with cofactor proteins (9, 18), often linked with estrogen-independent activation mediated by growth factor signaling pathways and mutations in the ERα (19). For example, overexpression or amplification of the steroid receptor coactivator-3 (SRC-3/NCOA-3/AIB1) has been associated with enhancement of ERα ligand-independent transactivation and poor prognosis in ERα positive breast cancer (20, 21, 22, 23). In contrast, it has also been shown that a reduction of ERα corepressors levels, such as Nuclear Receptor Corepressor 1 (NCOR1), leads to tamoxifen resistance (24, 25). Furthermore, the aberrant recruitment of nuclear cofactors has been linked with enhanced growth factor signaling altering the ERα phosphorylation state (26).

The interest in deciphering the landscape of ERα associated proteins within the disease state, has led to the development and improvement of several proteomic approaches. These include Rapid Immunoprecipitation Mass spectrometry of Endogenous proteins (RIME), which enables the identification of proteins crosslinked with a protein of interest by immunoprecipitation. This approach revealed an extensive set of proteins involved in ERα-mediated gene expression in human breast cancer cells and tissues as well as the switch between activation and repression of transcription in response to hormone therapy (27, 28). Although RIME identifies protein complexes, it does not provide details on the local environment of such proteins which may affect the binding of transcription factors to nearby DNA (29). Moreover, traditional protein-interaction methods such as Tandem Affinity Purification (TAP) coupled with quantitative Mass Spectrometry (MS) have been used to elucidate the ligand-activated (30) and RNA-mediated (31) ERα interactome. Although these immunoprecipitation-based approaches revealed several ERα molecular partners, they often result in the copurification of contaminant proteins and fail to capture transient interactions.

In the last decade, Proximity Labeling (PL) has enabled the dissection of challenging interactomes with a remarkable spatial and temporal resolution, transforming the interaction proteomics field (32). PL relies on enzymes genetically fused to a protein of interest that catalyze the covalent biotinylation of endogenous proximal proteins in living cells. Biotinylated proteins are subsequently enriched using streptavidin beads and identified by MS. Although over the past years a diverse set of enzymes have been engineered and successfully employed in a wide range of PL applications, including nuclear hormone receptors (33, 34), protein fusion is not always possible or desirable (35). Biotinylation by Antibody Recognition (BAR) was developed to overcome this limitation of traditional PL approaches. BAR replaces fusion enzymes with horseradish peroxidase (HRP)-conjugated antibodies to target a protein of interest and biotinylate neighbors upon the addition of a biotin substrate and hydrogen peroxidase (36). Advantages of antibody-based PL include avoiding fusion gene expression or CRISPR knock-in strategies for each target protein and capturing transient interactions in their spatial context. Importantly, BAR has been successfully employed to characterize endogenous protein interactions in primary human tissues (37). Therefore, this method offers the potential to translate protein interaction features from cells to clinical tumor samples. Since other studies previously captured ERα associated proteins using traditional PL methods (38, 39), we sought to investigate whether BAR can be used to map the ERα proximal proteome and its perturbations associated with acquired resistance to estrogen deprivation using aromatase inhibitors, one of the most widely used endocrine therapy approaches.

Here, we provide a solid workflow to target ERα using BAR. We assessed the specificity of the 6F11 endogenous antibody to target ERα and drive the biotinylation of its proximal proteins, comparing its performance with epitope antibody-mediated biotinylation. We further expanded our analysis to Long Term Estrogen Deprived (LTED) cells harboring both wildtype and mutated ERα (Y537C) modeling relapse on aromatase inhibitors (40, 41). Overall, this study shows that BAR is a valid method to explore the ERα proximal proteome and reveals intriguing alterations associated with adaptation to endocrine resistance.

## Experimental Procedures

### Cell Culture

MCF7-wt were purchased from the ATCC and cultured in phenol red-free RPMI supplemented with 10% Fetal Bovine Serum (FBS) and exogenous estradiol (1 nM). The respective Long Term Estrogen Deprived (LTED) derivatives harboring ERα^wt^ or a naturally occurring mutation ERα^Y537C^ which renders them ligand-independent were derived from parental cell lines as previously described (42). LTED cells were cultured in phenol red-free RPMI supplemented with 10% dextran charcoal stripped FBS (40). MCF7-Tet-Off (MCF7-TO) cells stably expressing ERα-Flag were kindly provided by Simak Ali (Imperial College London, United Kingdom) (43) and cultured in DMEM supplemented with 10% FBS, containing 100 mg/ml G418, 80 mg/ml Hygromycin B. Cells were treated with 1 μg/ml doxycycline for 24 h to silence the exogenous expression of ERα-Flag.

### Biotinylation by Antibody Recognition

BAR protocol was adapted from a previously published study (36). MCF7 cell lines were cultured in their respective basal medium and were not stripped of steroids prior to each experiment. Cells were grown in 15 cm dish to about 90% confluency, fixed in 4% Formaldehyde for 10 min, permeabilized in 0.5% Triton X-100 for 7 min and treated with H_2_O_2_ to inactivate the endogenous peroxidases for 10 min. After 1 h blocking in 1% BSA in PBST, cells were incubated overnight at 4 °C with the following primary antibodies: anti-ERα (#NCL-L-ER- 6F11 Novocastra), anti-Flag (#F1804 Sigma-Aldrich) and anti-tubulin (#T9026 Sigma). Samples were washed for 1 h in PBST and incubated with the secondary HRP-conjugate antibody (#P0447 Dako) for 1 h at room temperature (RT). Cells were washed for 1 h with PBST and resuspended in labeling buffer (140 μM Biotin phenol, 0.03% H_2_O_2_ in PBST). After 3 min, the biotinylation reaction was quenched by washing three times with 500 mM sodium ascorbate and subsequently with PBST. Biotinylation was confirmed by immunofluorescence on aliquot samples. For the affinity purification with streptavidin beads, cells were lysed in 2% SDS in PBST for 1 h at 95 °C. Protein extracts were quantified using BCA assay (Thermo). Lysates were diluted in PBST and incubated with pre-washed streptavidin magnetic beads (Thermo) for 2 h at RT (200 μl beads for 1 mg of total lysate). Beads were then washed once with PBST, twice with 1 M NaCl in PBST and twice with PBS. Biotinylation enrichment was assessed by western blot on beads aliquots.

### Immunofluorescence

Cells grown on coverslips were fixed in 4% Formaldehyde diluted in PBST for 10 min, permeabilized in 0.5% Triton X-100 for 7 min and blocked with 1% BSA in PBST before incubation with the following primary antibodies: anti-ERα (#NCL-L-ER- 6F11 Novocastra), anti-Flag (#F1804 Sigma-Aldrich) and anti-tubulin (#T9026 Sigma). Samples were then washed in PBST three times and incubated with secondary antibodies in blocking solution for 30 min. Secondary antibodies used were: Streptavidin Alexa Fluor 488 (#A32723 Thermo), Streptavidin Alexa Fluor 647 (#S32357 Thermo), Donkey anti-goat Alexa 594 (#705-585-147 Jackson ImmunoResearch). Cells were then washed in PBST three times and counterstained with 4,6-diamidino-2-phenylindole (DAPI). Coverslips were mounted on glass slides using Vectashield mounting medium (Vector Laboratories) and digital images were captured on a Zeiss LSM 710 confocal microscope or on a Zeiss spinning disk confocal microscope. Images were analyzed using Fiji imager software.

### Western Blot

After washing beads as described in ‘BAR’, biotinylated proteins were eluted incubating beads in SDS protein loading buffer with 2 mM biotin and 20 mM dithiothreitol for 10 min at 95 °C.

For total protein extraction, cells were lysed in extraction buffer (1% (v/v) Triton X-100, 10 mM Tris-HCl pH 7.4, 5 mM EDTA, 50 mM NaCl, 50 mM sodium fluoride, 2 mM Na_3_VO_4_, Complete inhibitor mix (Roche Applied Science) and homogenized by passage through a 26-gauge needle six times. The lysate was incubated on ice for 5 min and then clarified by centrifugation (14,000 rpm for 10 min at 4 °C). The protein concentration was then quantified with Bradford assay (Bio-Rad). Lysates were resolved by SDS-PAGE (Invitrogen) and transferred onto a nitrocellulose membrane (Amersham) for immunoblot analysis. After 1 h blocking with 5% milk in TBST at RT for 1 h, membranes were incubated with primary antibodies overnight at 4 °C. Following antibodies were used: anti-ER F10 (#sc-8002 Santa Cruz Biotechnology), streptavidin HRP conjugate (#S911 Thermo Scientific) anti-Flag (#F1804 Sigma-Aldrich) and tubulin (#T9026 Sigma).

### MS Sample Preparation

After three washes with 100 mM Triethylammonium bicarbonate (TEAB, Sigma) buffer, streptavidin beads were reduced by adding tris(2-carboxyethyl)phosphine (TCEP, Sigma, 20 mM final concentration) for 10 min at RT and alkylated with iodoacetamide (#I1149 Sigma, 12 mM final concentration) for 45 min at RT in the dark. Samples were then digested with 2 μg of trypsin (Mass Spectrometry Grade, ThermoFisher) overnight at 37 °C. Tryptic digests were transferred into fresh tubes after an additional incubation with 2 μg of trypsin for 3 h at 37 °C. Beads were further washed two times with 100 mM TEAB and all eluates combined.

For label-free analysis, samples were filtered using Pierce cellulose acetate spin filters (0.45 μm, ThermoFisher) and dried in a SpeedVac. For Tandem Mass Tag (TMT)-labeling, tryptic peptides were collected and labeled with TMTpro-16 plex reagent (ThermoFisher) according to manufacturer’s instructions. Labeled peptides were resuspended in 0.1% ammonium hydroxide/100% water and fractionated with high-pH Reversed-Phase chromatography on a Dionex UltiMate 3000 HPLC system using an XBridge C18 column (2.1 × 150 mm, 3.5 μm, Waters) at pH 10, with 30 min linear gradient from 5 to 35% acetonitrile (ACN)/ammonium hydroxide at a flow rate of 200 μl/min. The fractions were collected every 42 s, concatenated into 8 fractions and dried in a SpeedVac. The peptides were redissolved in 0.5% Formic Acid (FA) before liquid chromatography tandem mass spectrometry (LC-MS/MS) analysis.

### LC-MS/MS Analysis

The LC-MS/MS analysis was performed on a Orbitrap Fusion Lumos hybrid mass spectrometer coupled with an Ultimate 3000 RSLCnano UPLC system (ThermoFisher Scientific).

For label-free analysis, samples were first loaded and desalted on a PepMap C18 nano trap (100 μm i.d. × 20 mm, 100 Å, 5 μm). Then, peptides were separated on a PepMap C18 column (75 μm i.d. × 500 mm, 2 μm) over a linear gradient of 4 to 32% ACN/0.1% FA in 90 min at a 300 nl/min flow rate, total cycle time of 120 min for each fraction. The MS acquisition used standard data dependent acquisition (DDA) method with the Top Speed at 3 s per cycle time. The Orbitrap full MS scans (m/z 375–1500) were acquired at a resolution of 120,000 at m/z 200 and the automatic gain control (AGC) was set at 400,000 with 50 msec maximum injection time. The most abundant multiply charged ions (z = 2–5) with intensity above 8000 counts were subjected to MS/MS fragmentation by collision-induced dissociation (30% normalized collision energy) and detected in an ion trap for peptide identification. The isolation window was set at 1.6 Th and AGC at 10,000 with 50 msec maximum injection time. The dynamic exclusion window was set at 30 s with ±10 ppm.

For TMT-labeled samples, peptides were first loaded to a PepMap C18 nanotrap (100 μm i.d. × 20 mm, 100 Å, 5 μm) for 8 min at 10 μl/min with 0.1% FA/H_2_O, and then separated on a PepMap C18 column (75 μm i.d. × 500 mm, 100 Å, 2 μm) at 300 nl/min with a 90 min linear gradient of 4 to 30% ACN/0.1% FA in 90 min, total cycle time of 120 min for each fraction. The data acquisition used standard TMT MS2 based method with a cycle time at 3 s. The full MS scans (m/z 375–1500) were acquired in Orbitrap with a resolution at 120,000 at m/z 200 and AGC was set at 400,000 with maximum injection time at 50 msec. The most abundant multiply charged ions (2+ ∼ 6+) above intensity threshold at 10,000 were isolated by quadrupole at the isolation window at 0.7 Da and then subjected to MS/MS fragmentation by higher energy collisional dissociation (HCD) (36% normalized collision energy) and detected in the Orbitrap with the defined first mass at m/z 100, where resolution was set at 50,000, the AGC at 100,000 and maximum injection time at 86 msec. The dynamic exclusion range was set at 45 s with a ±7 ppm exclusion window.

### MS Data Processing

The raw files were processed with Proteome Discoverer 2.4 (ThermoFisher Scientific) using the Sequest HT search engine to search against the reviewed Uniprot database of Homo Sapiens (Version March 2021, total number of sequences were 20,324).

Search parameters for label-free samples were set as follow: trypsin full cleavage with two maximum miss-cleavage sites, mass tolerances at 20 ppm for the precursor, and 0.5 Da for the fragment ions; dynamic modifications of Deamidated (N, Q), Oxidation (M) and acetylation (Protein N-terminus), and static modification of Carbamidomethyl (C). For TMT samples parameters were: trypsin full cleavage with maximum two miss-cleavage sites, mass tolerances at 20 ppm for the precursor, and 0.02 Da for the fragment ions; dynamic modifications of Deamidated (N, Q), Oxidation (M), Phosphorylation (S, T, Y) and Acetylation (Protein N-terminus), and static modifications of TMTpro (K, Peptide N-terminus) and Carbamidomethyl (C).

Search result was validated by Percolator with q-value set at 0.01 for the decoy database search, and only high confident PSMs (Peptide Spectrum Matches) were considered. Protein False Discovery Rate (FDR) was set at 0.01. Only master proteins were reported. In the label-free sets, protein quantification used precursor intensity-based quantification and only unique peptides were considered for quantification. For reporter ion intensity detection in the TMT samples, the reporter ion quantifier node parameters were integration window tolerance 15 ppm, integration most confident centroid for peak detection and only unique peptides were considered for quantification. TMTpro’s Quan value correction factor, provided by the manufacturer’s certificate of analysis, was applied. Co-isolation threshold was set at 100, reporter ions average S/N threshold at 3.

### Data Analysis

Data filtering and statistical analysis were performed with Perseus (version 1.6.14.0) (44).

For ERα-Flag BAR, Label-Free Quantification (LFQ) values were log_2_ transformed and missing values imputed from normal distribution at the detection limit of the mass spectrometer using default parameters in Perseus. Values were median-normalized and statistically significant proteins were defined using a student’s *t* test (FDR < 0.05) comparing ERα-derived biotinylation and negative control samples. Only proteins with log_2_ fold change higher than 0.5 were considered significant and used for downstream analysis.

For ERα-wt BAR, LFQ values were log_2_ transformed and missing values replaced with the lowest value detected in the dataset. Statistically significant proteins were defined using a student *t* test (FDR < 0.05) comparing ERα-derived biotinylation and negative control samples. Only proteins with log_2_ fold change higher than 6 were considered significant and used for downstream analysis.

To assess the presence of false positives, a Receiver-Operating Characteristic (ROC) analysis was conducted on both ERα-wt and ERα-Flag BAR datasets. A ROC analysis shows the performance of discriminatory attributes by plotting the True Positive Rate (TPR) against the False Positive Rate (FPR) under various thresholds. The performance of the discriminatory parameter is measured as the area under the curve (AUC).

True Positive (TP) list included ERα known interactors from BioGRID (45) (physical interactions) and RIME (28, 46) datasets, together with human proteins with Gene Ontology (GO) Cellular Component (CC) terms containing nucleus (GO:0005634) and chromatin organization (GO:0006325) annotations. False Positive (FP) lists included human proteins with GOCC terms containing cytoplasm (GO:0005737), mitochondrion (GO:0005739) cell cortex (GO:0005938), endoplasmic reticulum (GO:0005783) and cell surface (GO:0009986).

TPR at different thresholds was computed using the following equation:

(1) $$TPR_{t}=\frac{T_{t}}{n}$$

Sup Equation 1. True positive rate. T is the number of TP annotated with a score below threshold (t), and n is the total number of TP.

FPR at various thresholds was computed using the following equation:

(2) $$FPR_{t}=\frac{F_{t}}{n}$$

Sup. Equation 2. False positive rate. F is the number of FP annotated with a score below threshold (t), and n is the total number of FP.

Optimal cut-off values were determined using the Youden’s J statistic, which captures the performance of a dichotomous diagnostic test.

(3) $$J=\frac{TP}{TP+FP}+\frac{TN}{TN+FP}-1$$

Sup. Equation 3. Youden's J statistic. TP is the number of True Positives, FP the number of False Positives and TN are the True Negatives.

For BAR on MCF7-wt, MCF7-LTED^wt^ and MCF7-LTED^Y537C^ analysis, TMT intensities in each channel were first divided by the median intensity of the corresponding TMT channel to normalize values across the TMTpro-16 plex. Next, each protein abundance was divided by the mean signal intensity of each gene across all samples and log_2_ transformed. Only proteins found in ERα BAR with intensity higher than tubulin BAR and control BAR were kept for further analysis. Additionally, we filtered out proteins detected with less than two unique peptides, to obtain a list of ERα putative proximal proteins. Student *t* test analysis (FDR < 0.05) comparing ERα-derived biotinylation and tubulin-derived biotinylation was performed to further confirm the validity of this filtering approach.

The pathway analysis and gene ontology enrichment analyses were performed by using the open-access bioinformatic tool g:profiler to determine biological pathways and categories significantly over-represented in selected protein lists (47). The network of proteins enriched in Reactome pathway terms was visualized with the Cytoscape v3.9.1 software (48).

### Experimental Design and Statistical Rationale

For ERα-wt and ERα-Flag BAR datasets, experiments were performed in three biological replicates (n = 3) and label-free quantification was used to assess the enrichment of biotinylated proteins compared to the negative control. A Flag-tag monoclonal antibody was used to target ERα in cells stably expressing exogenous ERα-Flag under the control of a Tet-Off promoter (MCF7-TO cells). Cells treated with doxycycline to silence the exogenous ERα expression were used as negative control. In MFC7-wt cells, the 6F11 monoclonal antibody targeting the ERα protein full length was used to convey the HRP antibody *in situ*. Primary antibody was omitted in control samples.

To investigate ERα proximal proteome rewiring associated with endocrine resistance, BAR experiments were performed targeting the endogenous ERα in MCF7-wt, MCF7-LTED^wt^ and MCF7-LTED^Y537C^ cell lines. BAR targeting tubulin was included for spatial specificity and primary antibody was omitted in control samples. In each cell line, experiments were performed in two independent replicates for ERα and tubulin (n = 2) and one replicate for the control samples (n = 1). TMT-based quantification was used to assess the enrichment of biotinylated proteins compared to the controls. Data processing and statistical analyses for each BAR experiment are described in detail previously.

## Results

### Antibody-Based Proximity Labeling Accurately Maps the ERα Proximal Proteome

**Fig. 1:**
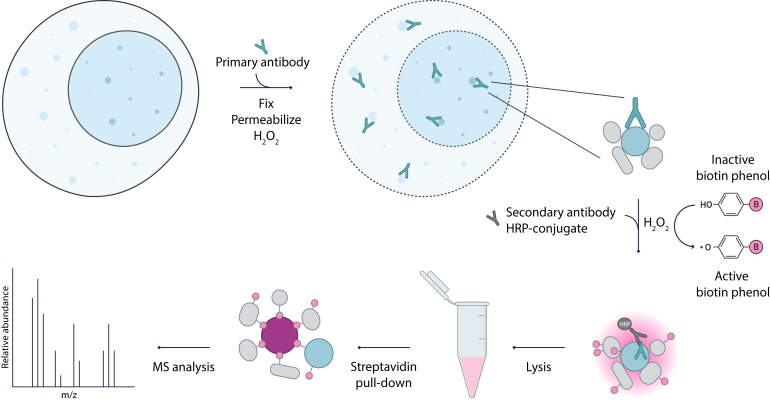
**BAR experimental workflow.** Illustration of BAR workflow. After fixation and permeabilization, cells are treated with hydrogen peroxide (H_2_O_2_) to inactivate the endogenous peroxidases. Then, cells are incubated with a primary antibody against the target protein and subsequently with a secondary HRP-conjugated antibody. The HRP peroxidase catalyzes the proximity labeling reaction upon the addition of biotin phenol and H_2_O_2_ producing free biotin phenol radicals that tag with biotin protein neighbors. Finally, biotinylated proteins are enriched using streptavidin beads and identified by MS.

**Fig. 2:**
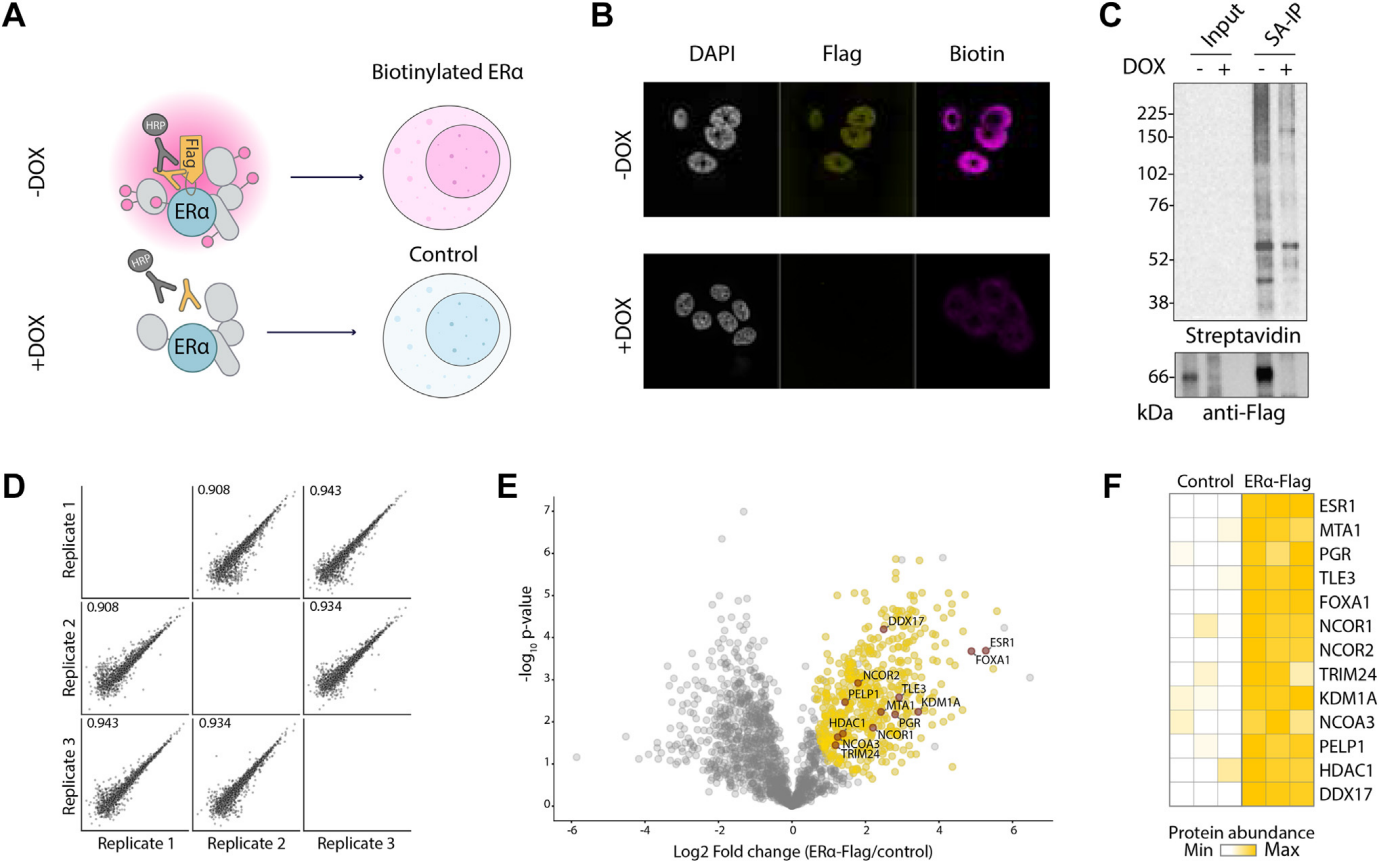
**Targeting the ERα proximal proteome using epitope tag antibody.***A*, schematic of BAR workflow targeting ERα-Flag. Cells stably expressing ERα-Flag under the control of a Tet-Off promoter (MCF7-TO cells) were labeled with biotin using epitope tag antibody to vehicle the proximity reaction. Cells were treated with doxycycline (DOX) to silence ERα-Flag expression in control samples. *B*, immunofluorescence images of ERα-Flag BAR. MCF7-TO cells treated with or without DOX were biotinylated as in (*A*). Biotin colocalized with ERα-Flag and biotinylation resulted in nuclear localization. Little biotinylation was observed in the negative control. Nuclei were detected by DAPI (*gray*); ERα-Flag was visualized by Alexa Fluor 488 dye (*gold*). Biotinylated proteins were detected by streptavidin-conjugated Alexa Fluor 647 dye (*magenta*). *C*, western blot analysis of ERα-Flag BAR biotinylated proteins. Labeled cells were lysed and incubated with streptavidin beads. ERα-Flag was efficiently immunoprecipitated (SA-IP) among biotinylated proteins (Input). *D*, multi scatter plot of proteins identified in the ERα-Flag BAR experiment. The log_2_-transformed label-free quantification intensities are plotted against each other. Pearson correlation coefficients for each comparison are reported in the upper left corner of the plots. *E*, volcano plot showing proteins significantly enriched in biotinylated *versus* control samples. Statistically significant proteins (student *t* test q-value <0.01) are shown in *gold*; proteins nonstatistically significant are shown in *gray*. ERα well-known interacting proteins are highlighted. *F*, heatmap showing ERα known interactors enriched *versus* negative control. Proteins are colored according to their abundance.

To address whether BAR was a suitable approach to explore the ERα proximal proteome, we first benchmarked the method targeting ERα with an epitope tag antibody in the human ERα positive breast cancer cell line MCF7. An overview of the workflow is illustrated in Figure 1*A*. Cells were first fixed and permeabilized to allow antibodies to access intracellular targets. Then, a primary antibody was used to target the protein of interest and an HRP-conjugate secondary antibody used to convert the inactive biotin substrate (biotin phenol) into a reactive biotin-intermediate, in the presence of H_2_O_2_. Once activated, highly reactive biotin radicals generated by the HRP catalysis diffuse away from the enzyme active site and, within minutes, covalently label tyrosine residues on proximal proteins typically in a radius of 10 to <200 nm (49). Biotin-tagged proteins were then identified by MS upon enrichment with streptavidin beads. In this setting, we employed cells stably expressing ERα-Flag under the control of a Tet-Off promoter (MCF7-TO cells) and used a monoclonal anti-Flag antibody to direct the proximal biotinylation. Importantly, the Tet-Off system provided the unique advantage of enabling gene expression silencing in the presence of doxycycline (DOX), thus representing a robust negative control for our experiments (Fig. 2*A* and supplemental Fig. S1, *A* and *B*).

We first tested biotinylation efficiency and specificity performing BAR in cells treated with or without DOX. Immunofluorescence (IF) revealed that the biotinylation pattern accurately recapitulated ERα nuclear localization in the absence of DOX. In contrast, little or no biotinylation was detected when ERα-Flag expression was silenced upon DOX treatment (Fig. 2*B*). We next assessed whether ERα-Flag was biotinylated and enriched upon streptavidin pull-down using lysates that were heat treated to reverse the crosslinking. Upon stringent washes, biotinylated proteins were eluted and analyzed by immunoblot. We found that ERα was specifically enriched among the biotinylated proteins compared to the negative control (Fig. 2*C*).

To determine whether we could effectively identify ERα proximal proteins using BAR, we performed MS analysis on three independent biological replicates of each condition. Biotinylated proteins were digested on the beads with trypsin and analyzed by MS. Using label-free quantification, we identified a total of 1929 proteins with high reproducibility between the three replicates (Fig. 2*D*). To remove non-specific background, we defined proteins significantly enriched in the biotinylated *versus* negative control samples using a student *t* test. This resulted in a list of 525 ERα-proximal protein candidates (q-value < 0.01, log_2_ fold change higher or equal to 0.5, supplemental Table S1) that included known interactors and putative novel proteins (Fig. 2*E*). Gene Ontology (GO) cellular component analysis of the ERα proximal protein candidates revealed a remarkable enrichment in nucleoplasm and chromatin, confirming the spatial resolution of the approach (supplemental Fig. S2*A*). Comparing our data with known ERα binding partners reported in the BioGRID protein interactions repository (45) (physical interactions) and ERα qPLEX-RIME dataset (28) (log_2_ fold change (ER/IgG) higher or equal to 1.2), BAR efficiently detected 174 proteins previously identified in the BioGRID and qPLEX-RIME datasets. These included several well-known ERα interactors such as FOXA1, NCOA3, HDAC1, DDX17, TRIM24, NCOR1, NCOR2, KDM1A and PELP1 (Fig. 2*F*). Thus, providing evidence that antibody-dependent biotinylation is a reliable method to map ERα proximal partners. Moreover, BAR detected 168 proteins not previously identified (supplemental Fig. S2*B*) comprising several chromatin regulators and transcription factors, which are known for their involvement in ERα regulation. Thus, suggesting that antibody-dependent biotinylation is a valid system to map the ERα proximal interacting partners, capturing not only compartment-specific proteomes but also direct binders.

### Comparing Efficiency and Specificity of Epitope Tagging *versus* Endogenous Antibodies for BAR

Next, we performed BAR using an endogenous antibody to target ERα in MCF7-wt cells. To achieve specific biotinylation we used a monoclonal antibody specific for the full length of ERα (clone 6F11), currently recognized as one of the most specific in evaluating the status of breast carcinomas (50). We benchmarked the specificity of the assay using the dataset from the epitope tag construct reported above.

**Fig. 3:**
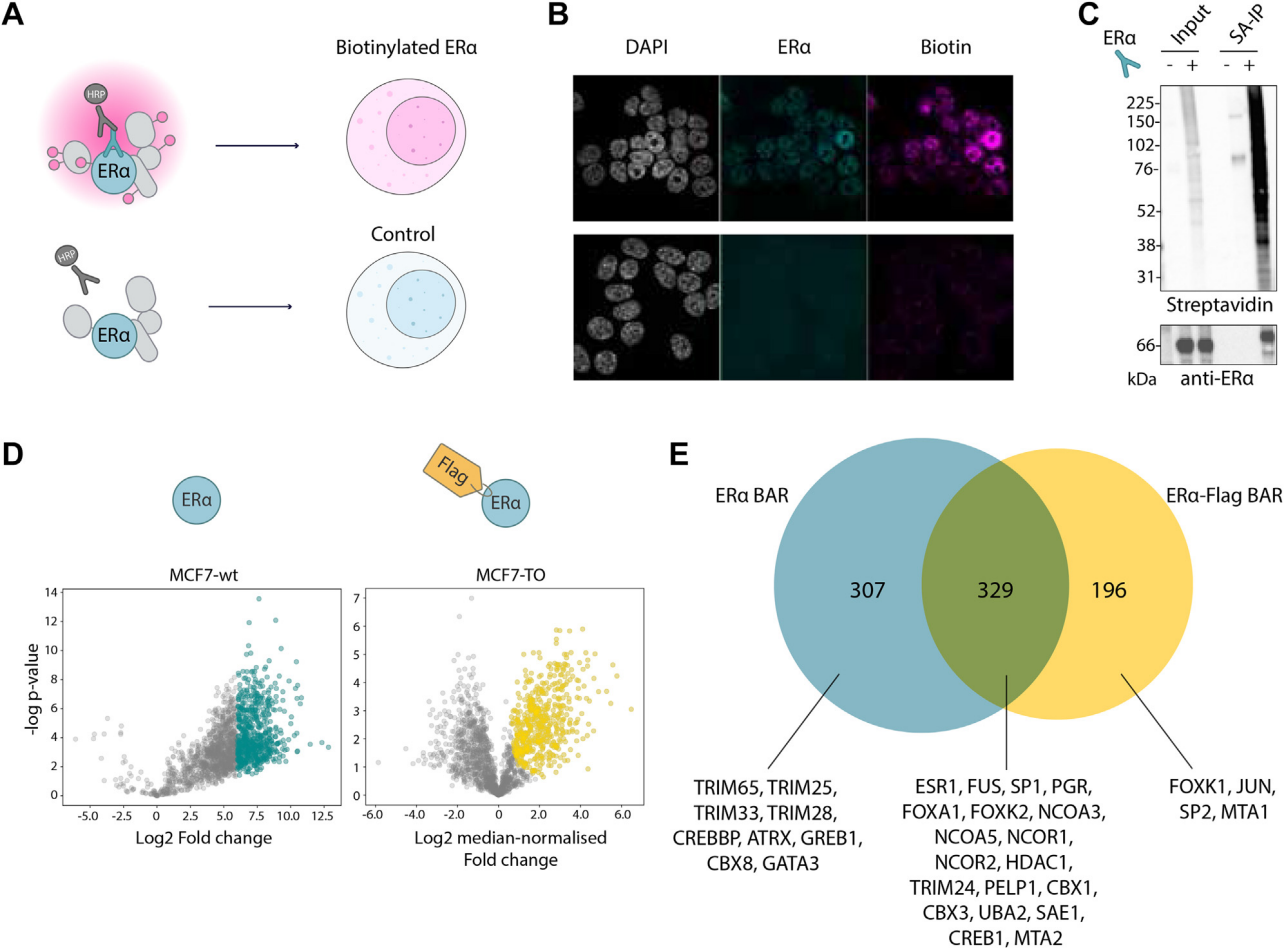
**ERα proximal proteins identified using the 6F11 monoclonal antibody.***A*, schematic of BAR workflow targeting endogenous ERα. A monoclonal 6F11 antibody was used to target ERα endogenously and drive the biotinylation of proximal proteins. Primary antibody was omitted in control samples. *B*, immunofluorescence images of ERα BAR biotinylation recapitulating endogenous antibody localization. Nuclei were detected by DAPI (*gray*); ERα was visualized by Alexa Fluor 488 dye (*cyan*). Biotinylated proteins were detected by streptavidin-conjugated Alexa Fluor 647 dye (*magenta*). *C*, streptavidin western blot analysis of ERα BAR biotinylation showing ERα enrichment upon streptavidin beads pull-down. *D*, volcano plots of biotinylated proteins identified in ERα (*right*) and ERα-Flag (*left*) BAR experiments. Significant proteins enriched over negative control are shown in *cyan* and *gold* for ERα for ERα-Flag BAR, respectively. Proteins nonstatistically significant are shown in *gray*. *E*, Venn diagram showing overlap between significant proteins identified in ERα and ERα-Flag BAR datasets.

First, we tested biotinylation specificity performing BAR in the presence or absence of the primary antibody (Fig. 3*A*). IF showed robust ERα antibody-dependent biotinylation reflecting nuclear protein localization comparable to tag-antibody staining. As expected, no biotinylation was observed in the negative control omitting the primary antibody (Fig. 3*B*). Similarly, western blot confirmed successful ERα enrichment compared to the negative control (Fig. 3*C*). Removing any reaction component (primary antibody, HRP-conjugate antibody, biotin phenol, H_2_O_2_) resulted in absence of biotinylation (supplemental Fig. S3, *A* and *B*).

Three biological replicates for each condition were analyzed by label-free MS. Out of 1830 proteins quantified in all three replicates, 636 were significantly enriched (student *t* test, q-value < 0.01, log_2_ fold change higher than 6, supplemental Table S2) in the biotinylated *versus* negative control samples (Fig. 3*D*, left panel in cyan). Nuclear proteins were successfully enriched upon removal of background binders (supplemental Fig. S5*C*). However, due to the lower levels of biotinylation in the control samples, these resulted in an overall higher number of missing values and consequent lower statistical power when removing background binders. Therefore, a more stringent fold change score was set as cutoff to discard non-specific binders compared to ERα-Flag BAR. This suggests that combining BAR with isobaric mass tags (such as Tandem Mass Tags (TMT) labels) is likely needed to overcome this drawback, especially when targeting low expression proteins. Nevertheless, a Receiver-Operating Characteristic (ROC) analysis on both datasets to assess the presence of false positives confirmed the cut-off values employed and provided additional confidence in the identification of *bona fide* proximal proteins (supplemental Fig. S4).

When compared to the Flag-derived biotinylation, 52% of the proteins identified using the endogenous antibody were also captured using the epitope tag antibody (Fig. 3*E*), including many classical ERα interacting proteins (*e.g.*, GREB1, FOXA1, NCOA3, NCOR1, HDAC1 and TRIM24). Moreover, several proteins were specifically detected using the 6F11 antibody (*i.e.*, GREB1, GATA3, CREBBP) or the epitope tag antibody (*i.e.*, JUN, FOXK1, SP2) as shown in Figure 3*E*. One possible explanation for this difference is that different antibodies may target distinct ERα complexes, potentially resulting in variations in the labeling of proximal proteins. However, another explanation is the difference in expression levels of ERα, which can also result in alternative proximal proteins. Remarkably, the endogenous antibody-based BAR (ERα BAR) confirmed 81 potential proximal proteins also detected using the tag antibody.

Comparison with published protein interaction datasets, which were manually curated and categorized based on the method employed for interaction detection (supplemental Table S3), revealed that nearly 42% of the ERα interactors identified with immunoprecipitation approaches (30, 31, 51, 52, 53) and 51% of the hits captured with RIME (28, 46) were also detected with BAR (supplemental Fig. S5, *A* and *B*). Particularly, 269 proteins identified in the ERα-wt BAR were previously reported in at least one other study, with 190 reported in multiple studies. When comparing the proteins identified by BAR with those detected by immunoprecipitation approaches reported in BioGRID, we observed a relatively limited overlap. This discrepancy can be attributed to various factors, including differences in experimental conditions, cell lines, methodologies, and mass spectrometry techniques. Noteworthy, there was also limited overlap among different ERα AP-MS datasets within BioGRID.

Among the ERα proximal protein candidates identified, BAR successfully captured several well-known direct interactors as well as 178 previously unreported proximal proteins. This demonstrates that the 6F11 antibody can effectively target endogenous ERα and label nearby proteins with biotin. Therefore, BAR serves as a valuable complementary approach, providing unique insights into proximal proteins that may not be captured by other methods. Importantly, since this approach avoids the use of external epitope tags, it provides a unique view of the unmodified ERα proximal proteome.

### Mapping the ERα Proximal Proteome Changes Associated with Endocrine Resistance Using BAR

**Fig. 4:**
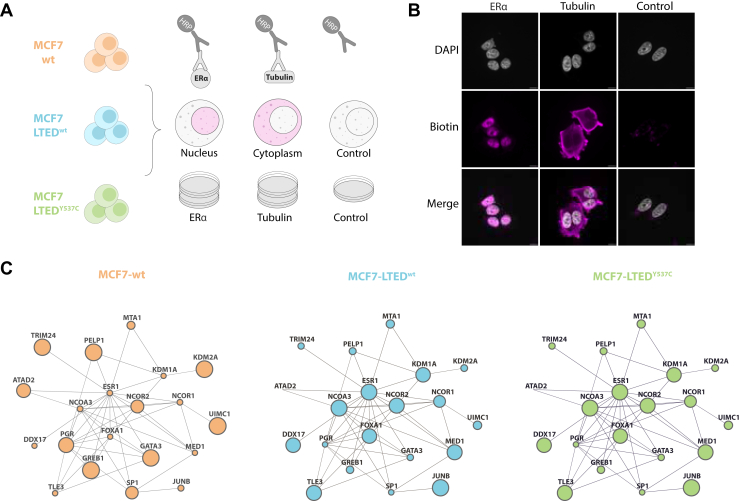
**Investigating the ERα proximal protein changes in cell modeling endocrine resistance.***A*, schematic of BAR experiments targeting the endogenous ERα in MCF7-wt, MCF7-LTED^wt^, and MCF7-LTED^Y537C^ cell lines. BAR targeting tubulin was used as spatial control to improve the enrichment of nuclear proteins. Primary antibody was omitted in control samples. Biotinylated proteins were enriched using streptavidin beads, protein digested, and peptides labeled with TMT before MS analysis. *B*, representative immunofluorescence images showing the different cellular compartment targeted with BAR in MCF7-wt cells. Nuclei were detected by DAPI (*gray*); Biotinylated proteins were detected by streptavidin-conjugated Alexa Fluor 647 dye (*magenta*). *C*, changes in ERα core-binding proteins in MCF7-wt, MCF7-LTED^wt^, and MCF7-LTED^Y537C^ cell lines detected using BAR. Node size is proportional to protein abundance.

Currently, one of the main challenges in breast cancer research is defining the ERα protein interaction perturbations associated with endocrine resistance. To investigate this, we used MCF7 Long Term Estrogen-Deprived (LTED) cells, a well-established isogenic cellular model that recapitulates the acquisition of aromatase inhibitor resistance in the clinic (40, 54). We performed a comparative analysis using BAR in combination with TMT quantitative MS investigating the differential ERα proximal proteome rewiring in MCF7-wt and MCF7-LTED cell lines. We also included resistant cells harboring a naturally occurring ERα^Y537C^ mutation modeling patients with metastatic tumors. To maximize the removal of ERα non-proximal binders, we included two different controls. First, we omitted the primary antibody to remove background proteins sticking to the beads. Second, to efficiently remove non-nuclear proteins we performed BAR targeting tubulin (spatial control), which improved the enrichment of nuclear proteins. We ran two biological replicates for ERα BAR and tubulin BAR as well as one replicate for control BAR for each cell line (Fig. 4*A*).

As expected, ERα BAR resulted in nuclear biotinylation and tubulin BAR showed cytoplasmic labeling. Negative controls revealed little or no biotinylation (Fig. 4*B*). To remove ERα non-proximal proteins from our dataset, we used a two-step filtering approach. First, to eliminate non-specific background we selected proteins showing higher average intensity in ERα BAR samples compared to the control BAR across the three cell lines. Then, we performed a similar comparison with Tubulin BAR samples to remove non-nuclear proteins. Using this approach, from the total of 4877 hits identified with MS, we refined a list of 975 ERα proximal protein candidates that specifically exhibited greater protein abundance in ERα BAR compared to the spatial reference and negative controls (supplemental Table S4). To cross-validate the refined dataset, we performed a student *t* test to identify proteins significantly enriched in ERα BAR compared to Tubulin BAR samples across the three cell lines (q-value <0.01). We found a substantial 72% overlap between the two approaches (supplemental Fig. S6*A*), supporting the validity and reliability of our filtering strategy. Furthermore, GO cellular components analysis confirmed enrichment in nuclear proteins after removing non-proximal proteins found in Tubulin BAR (supplemental Fig. S6*B*). Within the list of ERα proximal candidates, 257 proteins were also detected in the previous BAR experiments and 206 were previously identified in other ERα interactomics studies (28, 30, 31, 46, 51, 52, 53).

Assessment of the ERα proximal proteome changes associated with endocrine resistance revealed similarities and differences in the ERα signaling complex (Fig. 4*C*). In agreement with our previous quantitative proteomic analysis of MCF7-wt and the MCF7-LTED^wt^ cell lines (55), we found increased expression of ERα as well as enrichment of NCOA3, FOXA1, TLE3 and DDX17 in LTED derivatives. In contrast, several estrogen-regulated genes, including GREB1 and PGR, showed specific enrichment in estrogen-sensitive cells (supplemental Fig. S6*C*). These results were further supported by our previous qPLEX-RIME analysis comparing the three isogenic MCF7 models (56), which showed that TLE3 was the most enriched protein identified in MCF7-LTED^wt^ cells (supplemental Fig. S6*D*). Taken together, these data suggest that BAR efficiently captures important ERα proximal proteome alterations across cells modeling adaptation to endocrine resistance.

### Increased Levels of ERα Expression Impact Proximal Proteome Rewiring in Resistant Cell Lines

**Fig. 5:**
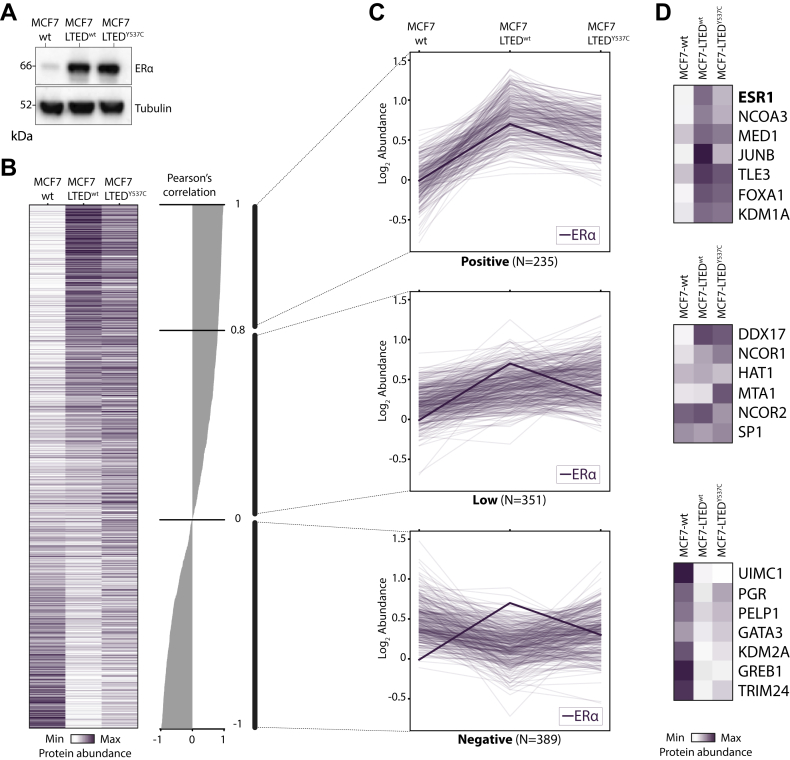
**ERα proximal protein clustering based on bait expression levels in cell line modeling endocrine resistance.***A*, western blot showing alterations in ERα expression levels associated with endocrine-resistant phenotypes. *B*, heatmap showing protein abundance changes of ERα proximal protein candidates identified with BAR in MCF7-wt, MCF7-LTED^wt^, and MCF7-LTED^Y537C^ cell lines. Proteins are ranked by Pearson’s correlation similarity to ERα abundance. Proteins are colored according to their abundance (average of duplicates). *C*, three clusters were generated based on Pearson’s correlation similarity scores between the ERα and its putative proximal protein abundances. Profile plots are shown for each cluster; ERα protein abundance profile is represented with a thicker line. *D*, abundance changes of ERα core-interacting proteins representative for each cluster.

MCF7-LTED cells upregulate ERα protein levels from five to seven fold to maximize the response to residual estrogen (54) (Fig. 5*A*). To investigate the impact of altered ERα expression levels on its proximal proteome, we employed Pearson’s correlation to determine the relationship between ERα expression and its proximal protein candidates (supplemental Fig. S7). In this setting, proteins that exhibit high correlation scores will be dependent on ERα levels rather than its proximal proteome rewiring. Using this approach, we defined three main clusters of proteins with positive, low and negative dependency on bait levels (Fig. 5*B*). Notably, despite BAR captures proximal proteomes rather than interactors, all three groups comprised previously reported ERα direct binders (Fig. 5*D*).

Proteins with positive correlation with ERα expression were specifically enriched in MCF7-LTED^wt^ cells, compared to both MCF7-wt and MCF7 LTED^Y537C^. These included several well-known ERα coactivators such as NCOA3, FOXA1, MED1, DDX17 and KDM1A, suggesting that ERα functions in a ligand-independent manner in resistant cell lines (Fig. 5*D*). Noteworthy, in this cluster we also found TLE3 and JUNB. JUNB belongs to the AP-1 transcription complex and it has been suggested to be a key determinant of endocrine resistance by mediating a global shift in the ERα transcriptional program influencing the expression of genes associated with resistance (57). On the contrary, proteins with low dependency on the bait levels showed overall distinct proximal proteome patterns compared to ERα levels. These included proteins with similar levels in the three cell models (*i.e.*, HAT1) or proteins enriched in LTED^Y537C^ cells (*i.e.*, MTA1). Interestingly, we found 389 proteins with negative correlation with ERα levels, whose changes were not directly driven by the bait expression. These included several ERα coregulators such as GREB1, GATA3, PGR, TRIM24, ATAD2, and PELP1 (Fig. 5*D*). GREB1 was previously shown to be an estrogen-specific ERα interacting protein and its expression is downregulated in multiple endocrine-resistant cell models (46, 58, 59). In this cluster, we also found the ubiquitin-binding protein UIMC1 (also known as RAP80) as the protein most strongly associated with ERα in MCF7-wt cells. In line with our data, UIMC1 was shown to interact with ERα and modulate its ubiquitination in the presence of estrogen but not in the presence of antagonists, such as fulvestrant and tamoxifen (60). Importantly, this cluster showed comparable protein signatures between MCF7-wt and MCF7-LTED^Y537C^ cells, in agreement with our previous RIME data and confirming prior reports (40). Together, these findings show that changes in proximal proteome correlate with global levels of ERα in acquired endocrine resistant phenotypes.

### ERα Proximal Protein Interaction Network

**Fig. 6:**
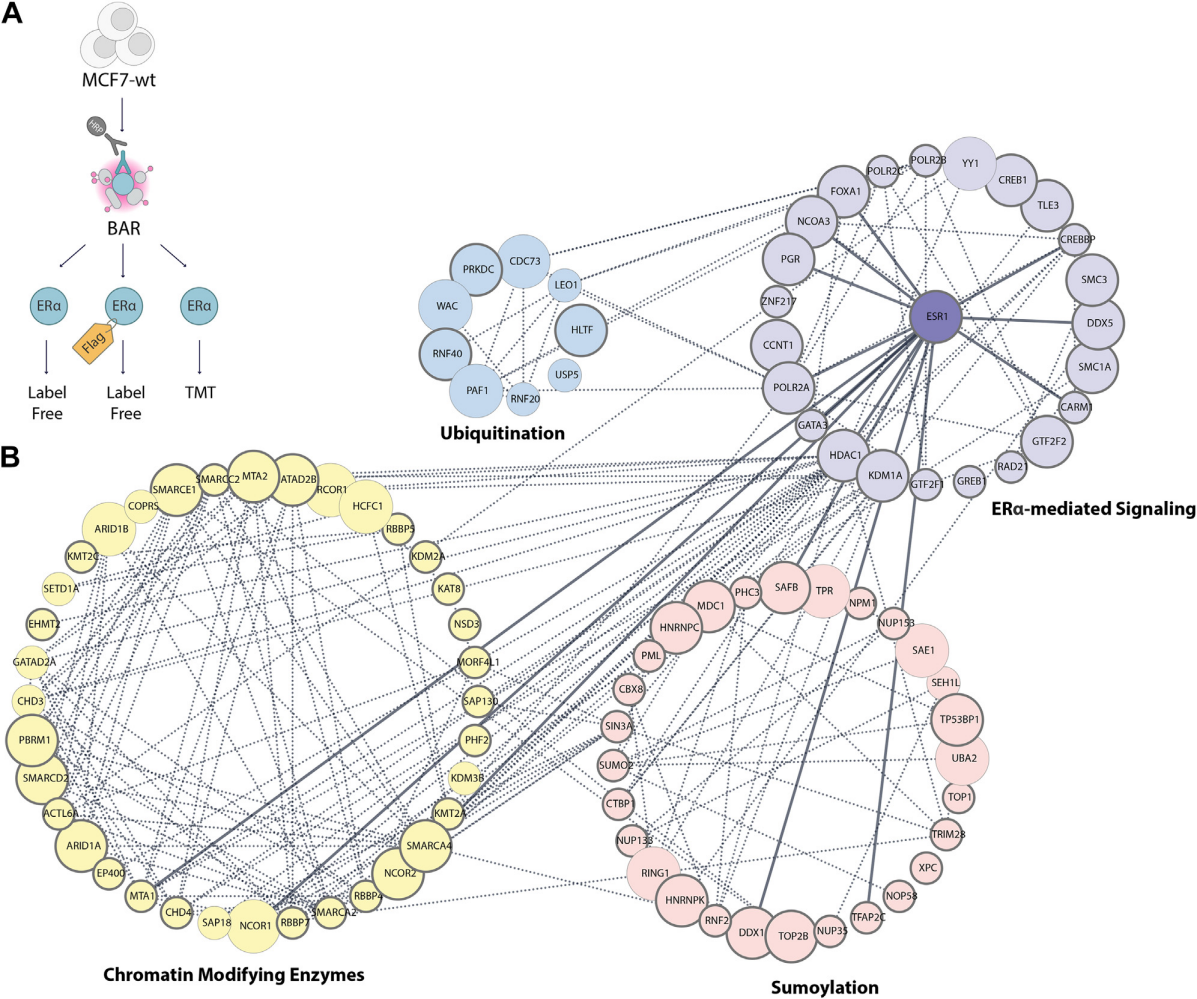
**ERα proximal protein network.***A*, schematic illustration of the integrated data obtained from each independent BAR experiment. Proteins significantly enriched over the respective negative control in ERα-Flag BAR, ERα BAR, and ERα BAR TMT were combined to build a high-confidence ERα proximal protein network. Only proteins found in at least two out of three datasets were included in this analysis. *B*, network of the ERα proximity proteins organized by relevant biological processes. Individual proteins are shown as nodes and interactions as edges. Proteins are colored according to functional clustering. Bigger nodes indicate protein identified in three out of three independent BAR datasets. ERα first neighbor interactions are shown with thicker lines.

To provide a snapshot of the ERα proximal proteome, we built a protein interaction network combining three independent BAR datasets (Fig. 6*A*). This resulted in 461 high-confidence proximal protein candidates significantly enriched over the controls and found in at least two out of three BAR datasets (supplemental Table S5).

Notably, the vast majority (98%) of the candidates were annotated as nuclear, consistent with ERα subcellular localization. Furthermore, Reactome pathway analysis revealed the enrichment of several biological pathways, including the generic transcription pathway, sumoylation, chromatin modifying enzymes, epigenetic regulation of gene expression, estrogen-dependent gene expression, ERα-mediated signaling, signaling by nuclear receptors, and protein ubiquitination (full annotation list in Supplemental Table S6). GO biological processes analysis further confirmed the enrichment of several transcription and chromatin remodeling processes within the list of high-confidence ERα proximal protein candidates (full annotation list in Supplemental Table S6).

We next selected proteins involved in dynamic processes such as sumoylation and ubiquitination, as well as proteins involved in ERα biology including chromatin remodeling processes, to investigate transient interactions while reducing the complexity of the protein interaction network. This resulted in a refined set of high-confidence ERα proximal candidates consisting of 97 proteins (supplemental Table S5). A functional protein network was derived using known protein interactions from the STRING database (61) (protein edges with combined score higher than 0.4, Fig. 6*B*). Not surprisingly, the larger cluster within this network consisted of chromatin modifying enzymes, which is consistent with the role of ERα in chromatin remodeling mechanisms. Additionally, it is intriguing to observe that the second most represented proteins in the network were associated with sumoylation. While the role of sumoylation remains poorly understood in the context of breast cancer (62), it has been previously shown that estrogen resistant models harboring mutations in the LBD of ERα exhibit reduced sumoylation and consequent increased transcriptional activity (63). Thus, highlighting the potential significance of sumoylation machinery in breast cancer. Furthermore, we found that among the proteins identified in this network, 66 had previously been reported to interact with ERα using different methods (BioGRID interaction repository database (45) and qPLEX-RIME dataset (28)) (Fig. 6*B*, circled in dark gray). Interestingly, BAR detected an additional 22 proteins that were not previously reported in these datasets, suggesting potential novel ERα proximal proteins. Notably, 12 of these putative proximal partners were consistently detected in all three independent BAR experiments, (Fig. 6*B*, represented by bigger nodes). These included several components of the BAF complex, such as SMARCA4 (BRG1), as well as other chromatin binding proteins like HCFC1. Additionally, several transcription regulators, such as NCOR1 and CREB1, were also identified. Of particular importance, the network included sumo-activating enzymes, such as SAE1 and UBA2, which are often challenging to detect due to their transient nature.

We next investigated whether the refined ERα proximal proteome exhibited changes associated with acquired resistance. To address this, we mapped the protein clusters, which were defined based on ERα expression levels in both sensitive and resistant cell line models, onto the high-confidence proximal proteome (supplemental Fig. S8). We found that proteins involved in ERα-mediated transcription (*i.e.*, FOXA1, NCOA3, KDM1A) and components of the chromatin remodeling complex (*i.e.*, SMARCE1, SMARCD2, RCOR1) showed enhanced recruitment in resistant cell lines. Suggesting that increased expression of ERα in resistant cells facilitates the recruitment of these proteins, potentially leading to sustained transcriptional activity and promoting cell growth. In contrast, several coregulators (*i.e.*, GATA3, PGR, CREBBP, GREB1) involved in ERα-mediated transcription, exhibited specific recruitment in endocrine sensitive models. This suggests that development of resistance may disrupt interactions between ERα and canonical coregulators, leading to compromised transcriptional regulation and perturbed downstream signaling pathways. Of note, approximately 30% of the refined ERα proximal proteome exhibited changes that were not directly associated with ERα expression levels. Among these we found the previously unreported interaction with RING1, an E3 ubiquitin-protein ligase consistently identified in three independent BAR experiments. This protein remained consistent between sensitive and resistant models suggesting a functional role independent of the resistance phenotype.

Collectively, our BAR datasets provide a comprehensive snapshot of the ERα proximal proteome. Antibody-based proximity labeling successfully detected several known ERα interactors and efficiently identified proteins involved in dynamic processes such as sumoylation and ubiquitination, which are challenging to detect with traditional affinity purification PPIs approaches. Furthermore, our analysis revealed notable alterations in the ERα proximal proteome, including enhanced recruitment of specific proteins in resistant cells and the downregulation of distinct coregulators in the sensitive state. This comprehensive characterization of the ERα proximal proteome provides insights on the complexity of ERα-mediated signaling and its potential contribution to acquired resistance in breast cancer.

## Discussion

In this study, we describe biotinylation by antibody recognition as a valid approach to investigate the ERα proximal proteome in a spatial context. Furthermore, we evaluate BAR applicability to map the perturbed ERα proximal proteome in cells modeling endocrine resistance. Combining our three independent BAR experiments, we delineate an ERα proximal protein network and its changes associated with acquired resistance. Antibody-based PL not only successfully detected several known ERα interactors, but more importantly identified proximal proteins involved in dynamic processes such as ubiquitination and sumoylation that are challenging to detect with traditional PPIs approaches.

A critical step to successfully employ BAR is identifying a suitable antibody to target a protein of interest. Comparison of biotinylated protein patterns in ERα and ERα-Flag BAR experiments revealed that targeting ERα with the 6F11 monoclonal antibody resulted in similar efficiency to epitope tag. We show that both antibodies led to the successful identification of 329 known ERα interactors, including FOXA1, NCOA3, HDAC1, NCOR1, TRIM24, PELP1 and CBX3. Interestingly, despite a large number of candidate proteins being shared across ERα and ERα-Flag BAR datasets, some of the well-known ERα interactors (including GREB1 and GATA3) were only detected targeting the endogenous ERα (Fig. 3*E*). Thus highlighting the importance of targeting the endogenous protein and emphasizing the advantage of avoiding genetic manipulation often required in classical PPIs and PL methods.

On the other hand, ERα BAR resulted in overall a higher background compared to ERα-Flag. We believe this difference arises from the lower expression levels of the endogenous ERα (supplemental Fig. S1, *A* and *B*) and not from the antibody, given the large overlap between the two datasets with over 40% proteins detected in both experiments. High non-specific background also affects the results of immunoprecipitation experiments particularly when protein baits present low expression levels. However, combining AP-MS and PL has been recently shown to overcome these limitations (64). Another explanation for this discrepancy is the competitive advantage of using cells not expressing the target protein as negative control. In this setting, the addition of both primary and HRP-conjugated antibodies efficiently enables the removal of non-specific biotinylation originating from primary antibody stochastic binding. Therefore, for future BAR applications we suggest using knockout cells as a negative control where possible. Additionally, several protein silencing tools have been developed as valid alternatives, including RNA interference and targeted proteolysis. Importantly, our data show that spatial controls effectively allow the removal of proteins localized in specific cellular compartments similarly to ‘classical PL methods’. Unlike these approaches, in which protein biotinylation depends on its distance from the enzyme and the finite dwell time of activated biotin phenol (65), we found that biotinylation does not quantitatively reflect the relative localization from the enzyme given that BAR is restricted to fixed and permeabilized cells. Furthermore, it is important to note that HRP enzymes catalyze the oxidation of short lived biotin-phenol radicals that react with electron-rich amino acids, primarily tyrosine, on neighboring proteins resulting in their covalent tagging with biotin (66). Therefore, protein biotinylation efficiency will be affected by the number of accessible tyrosine residues.

Although BAR compared to traditional PPIs methods allows to perform more stringent washing conditions and remove non-specific binders, due to the nature of proximity labeling, the data generated will include bystander proteins. Therefore, designing the appropriate controls to obtain strong enrichment of proteins residing in the same cellular compartment is crucial (35, 36). We show that targeting a protein residing in the cytoplasm enables robust enrichment for nuclear ERα interactors with high specificity (supplemental Fig. S6*B*). For future experiments, we suggest targeting a protein localized in a different subcellular compartment with similar expression levels compared to the protein of interest as spatial control. Compared to other PPIs approaches, the ability to control for spatial specificity represents a significant advantage and provides subcellular context to comprehensively characterize protein interaction landscapes.

Our BAR data assessing the ERα proximal proteome changes in MCF7-wt, MCF7-LTED^wt^ and MCF7-LTED^Y537C^ cell lines revealed interesting alterations related to acquired resistance. We found that ERα is associated with several classical cofactors (*i.e.*, NCOA3, FOXA1, DDX17 and KDM1A) in resistant cells (Fig. 4*C*) and their association is directly related with ERα overexpression (Fig. 5*D*). Suggesting that the ERα functions in a ligand-independent manner in resistant models. ERα cofactors coordinate several signaling pathways and have been previously reported to play an important role in the development of resistance phenotypes (67, 68). As expected, we found that in estrogen sensitive cells ERα associates with hormone-regulated proteins. These include GREB1, GATA3, PGR, TRIM24, and PELP1 (Figs. 4*C* and 5D). Furthermore, the ubiquitin-binding protein UIMC1, previously shown to interact with ERα (60), was one of the most enriched proximal proteins in MCF7-wt cells compared to LTED derivatives (Fig. 4*C*). This finding on one hand confirms that BAR can capture weak interactions as ubiquitin and ubiquitin-binding domains (69). On the other hand, it suggests that the levels of ubiquitinated ERα are higher in sensitive cells compared to resistant models. In support of this hypothesis, it has been previously shown that the binding of different ligands to ERα affects the receptor stability and ubiquitination (70). In the same study, it was shown that in the presence of tamoxifen ERα was hypo-ubiquitinated and more stable. Thus suggesting that ERα ubiquitination and consequent degradation may be linked to endocrine resistance (71). In keeping with our previous data, several hormone-regulated proteins showed a similar signature in MCF7-LTED^Y537C^ compared to MCF7-wt cells (Fig. 5*C*, lower panel), suggesting that ESR1^Y537C^ functions in a ligand-dependent manner compared to MCF7-LTED^wt^ (40).

Combining data from three independent BAR experiments, we built an ERα proximal protein network. We refined a list of 97 high-confidence ERα proximal candidates by selecting proteins involved in dynamic processes such as sumoylation and ubiquitination, as well as proteins involved in ERα biology and chromatin remodeling processes. This network covers the vast majority of the ERα published interactors and their connections with novel partners. Of note, only a handful of ERα interactors (*i.e.*, STAT1, YAP and TEAD) found in other studies using traditional PL approaches (38, 39) were not detected in our analysis. However, differences in bait expression levels, as well as variations in the PL enzymes, engineered cell lines, and MS methods used, can account for some of the observed differences. As expected, our proximal protein network showed a strong link between ERα coregulators and proteins involved in chromatin remodeling processes and revealed 22 new proteins not previously reported to associate with ERα. These include proteins specifically recruited in resistant cells such as SETD1A, a key epigenetic regulator of ERα shown to play a role in promoting the survival of tamoxifen-resistant cells (72). Compared to existing ERα interactome datasets, BAR uncovered associations with several proteins involved in post translational modifications such as ubiquitination and sumoylation. An interesting example is represented by the proteins involved in ubiquitination processes. Only 3 out of 9 proteins involved in ubiquitination were previously detected (Fig. 6*B*, light blue cluster), highlighting the ability of BAR to detect transient interactions more efficiently compared to traditional approaches.

In summary, we demonstrate that BAR enables the identification of proteins involved in dynamic ERα interaction networks and uncovers protein proximity *in situ* thereby bypassing the preference of classical approaches for stable interactions. We provide strong evidence that the 6F11 monoclonal antibody enables effective antibody-based PL, opening exciting opportunities in the field to delineate the ERα proximal proteome adaptation in clinical samples.

## Data Availability

The mass spectrometry proteomics data have been deposited to the ProteomeXchange Consortium *via* the PRIDE (73) partner repository with the dataset identifier PXD043294.

## Supplemental Data

This article contains supplemental data (28, 30, 31, 46, 51, 52, 53, 56).

## Conflict of interest

The authors declare no competing interests.

## References

[1] Arnold M., Morgan E., Rumgay H., Mafra A., Singh D., Laversanne M. (2022). Current and future burden of breast cancer: global statistics for 2020 and 2040. Breast.

[2] Dodson A., Parry S., Ibrahim M., Bartlett J.M., Pinder S., Dowsett M. (2018). Breast cancer biomarkers in clinical testing: analysis of a UK national external quality assessment scheme for immunocytochemistry and *in situ* hybridisation database containing results from 199 300 patients. J. Pathol. Clin. Res..

[3] Tora L., White J., Brou C., Tasset D., Webster N., Scheer E. (1989). The human estrogen receptor has two independent nonacidic transcriptional activation functions. Cell.

[4] Chen D., Washbrook E., Sarwar N., Bates G.J., Pace P.E., Thirunuvakkarasu V. (2002). Phosphorylation of human estrogen receptor alpha at serine 118 by two distinct signal transduction pathways revealed by phosphorylation-specific antisera. Oncogene.

[5] Kato S., Endoh H., Masuhiro Y., Kitamoto T., Uchiyama S., Sasaki H. (1995). Activation of the estrogen receptor through phosphorylation by mitogen-activated protein kinase. Science.

[6] Weitsman G.E., Li L., Skliris G.P., Davie J.R., Ung K., Niu Y. (2006). Estrogen receptor-alpha phosphorylated at Ser118 is present at the promoters of estrogen-regulated genes and is not altered due to HER-2 overexpression. Cancer Res..

[7] Medunjanin S., Hermani A., De Servi B., Grisouard J., Rincke G., Mayer D. (2005). Glycogen synthase kinase-3 interacts with and phosphorylates estrogen receptor alpha and is involved in the regulation of receptor activity. J. Biol. Chem..

[8] Park K.-J., Krishnan V., O'Malley B.W., Yamamoto Y., Gaynor R.B. (2005). Formation of an IKKalpha-dependent transcription complex is required for estrogen receptor-mediated gene activation. Mol. Cell.

[9] Green K.A., Carroll J.S. (2007). Oestrogen-receptor-mediated transcription and the influence of co-factors and chromatin state. Nat. Rev. Cancer.

[10] Farcas A.M., Nagarajan S., Cosulich S., Carroll J.S. (2021). Genome-wide estrogen receptor activity in breast cancer. Endocrinology.

[11] Molehin D., Rasha F., Rahman R.L., Pruitt K. (2021). Regulation of aromatase in cancer. Mol. Cell. Biochem..

[12] Feng Q., O'Malley B.W. (2014). Nuclear receptor modulation--role of coregulators in selective estrogen receptor modulator (SERM) actions. Steroids.

[13] Nathan M.R., Schmid P. (2017). A review of fulvestrant in breast cancer. Oncol. Ther..

[14] Ma C.X., Reinert T., Chmielewska I., Ellis M.J. (2015). Mechanisms of aromatase inhibitor resistance. Nat. Rev. Cancer.

[15] Musheyev D., Alayev A. (2022). Endocrine therapy resistance: what we know and future directions. Explor. Target. Antitumor Ther..

[16] Jeselsohn R., Yelensky R., Buchwalter G., Frampton G., Meric-Bernstam F., Gonzalez-Angulo A.M. (2014). Emergence of constitutively active estrogen receptor-α mutations in pretreated advanced estrogen receptor-positive breast cancer. Clin. Cancer Res..

[17] Toy W., Shen Y., Won H., Green B., Sakr R.A., Will M. (2013). ESR1 ligand-binding domain mutations in hormone-resistant breast cancer. Nat. Genet..

[18] Métivier R., Penot G., Hübner M.R., Reid G., Brand H., Kos M. (2003). Estrogen receptor-alpha directs ordered, cyclical, and combinatorial recruitment of cofactors on a natural target promoter. Cell.

[19] Razavi P., Chang M.T., Xu G., Bandlamudi C., Ross D.S., Vasan N. (2018). The genomic landscape of endocrine-resistant advanced breast cancers. Cancer Cell.

[20] Lahusen T., Henke R.T., Kagan B.L., Wellstein A., Riegel A.T. (2009). The role and regulation of the nuclear receptor co-activator AIB1 in breast cancer. Breast Cancer Res. Treat..

[21] Heery D.M., Kalkhoven E., Hoare S., Parker M.G. (1997). A signature motif in transcriptional co-activators mediates binding to nuclear receptors. Nature.

[22] List H.J., Reiter R., Singh B., Wellstein A., Riegel A.T. (2001). Expression of the nuclear coactivator AIB1 in normal and malignant breast tissue. Breast Cancer Res. Treat..

[23] Anzick S.L., Kononen J., Walker R.L., Azorsa D.O., Tanner M.M., Guan X.Y. (1997). AIB1, a steroid receptor coactivator amplified in breast and ovarian cancer. Science.

[24] Haque M.M., Desai K.V. (2019). Pathways to endocrine therapy resistance in breast cancer. Front. Endocrinol. (Lausanne).

[25] Osborne C.K., Schiff R. (2011). Mechanisms of endocrine resistance in breast cancer. Annu. Rev. Med..

[26] Musgrove E.A. (2011). Estrogen receptor degradation: a CUE for endocrine resistance?. Breast Cancer Res..

[27] Mohammed H., Taylor C., Brown G.D., Papachristou E.K., Carroll J.S., D’Santos C.S. (2016). Rapid immunoprecipitation mass spectrometry of endogenous proteins (RIME) for analysis of chromatin complexes. Nat. Protoc..

[28] Papachristou E.K., Kishore K., Holding A.N., Harvey K., Roumeliotis T.I., Chilamakuri C.S.R. (2018). A quantitative mass spectrometry-based approach to monitor the dynamics of endogenous chromatin-associated protein complexes. Nat. Commun..

[29] van Mierlo G., Vermeulen M. (2021). Chromatin proteomics to study epigenetics - challenges and opportunities. Mol. Cell. Proteomics.

[30] Tarallo R., Bamundo A., Nassa G., Nola E., Paris O., Ambrosino C. (2011). Identification of proteins associated with ligand-activated estrogen receptor α in human breast cancer cell nuclei by tandem affinity purification and nano LC-MS/MS. Proteomics.

[31] Nassa G., Giurato G., Salvati A., Gigantino V., Pecoraro G., Lamberti J. (2019). The RNA-mediated estrogen receptor α interactome of hormone-dependent human breast cancer cell nuclei. Sci. Data.

[32] Dionne U., Gingras A.-C. (2022). Proximity-dependent biotinylation approaches to explore the dynamic compartmentalized proteome. Front. Mol. Biosci..

[33] Vélot L., Lessard F., Bérubé-Simard F.-A., Tav C., Neveu B., Teyssier V. (2021). Proximity-dependent mapping of the androgen receptor identifies Kruppel-like factor 4 as a functional partner. Mol. Cell. Proteomics.

[34] Lempiäinen J.K., Niskanen E.A., Vuoti K.-M., Lampinen R.E., Göös H., Varjosalo M. (2017). Agonist-specific protein interactomes of glucocorticoid and androgen receptor as revealed by proximity mapping. Mol. Cell. Proteomics.

[35] Cho K.F., Branon T.C., Udeshi N.D., Myers S.A., Carr S.A., Ting A.Y. (2020). Proximity labeling in mammalian cells with TurboID and split-TurboID. Nat. Protoc..

[36] Bar D.Z., Atkatsh K., Tavarez U., Erdos M.R., Gruenbaum Y., Collins F.S. (2018). Biotinylation by antibody recognition-a method for proximity labeling. Nat. Methods.

[37] Killinger B.A., Marshall L.L., Chatterjee D., Chu Y., Bras J., Guerreiro R. (2022). *In situ* proximity labeling identifies Lewy pathology molecular interactions in the human brain. Proc. Natl. Acad. Sci. U. S. A..

[38] Liu Z., Merkurjev D., Yang F., Li W., Oh S., Friedman M.J. (2014). Enhancer activation requires trans-recruitment of a mega transcription factor complex. Cell.

[39] Zhu C., Li L., Zhang Z., Bi M., Wang H., Su W. (2019). A non-canonical role of YAP/TEAD is required for activation of estrogen-regulated enhancers in breast cancer. Mol. Cell.

[40] Martin L.-A., Ribas R., Simigdala N., Schuster E., Pancholi S., Tenev T. (2017). Discovery of naturally occurring ESR1 mutations in breast cancer cell lines modelling endocrine resistance. Nat. Commun..

[41] Schiavon G., Hrebien S., Garcia-Murillas I., Cutts R.J., Pearson A., Tarazona N. (2015). Analysis of ESR1 mutation in circulating tumor DNA demonstrates evolution during therapy for metastatic breast cancer. Sci. Transl. Med..

[42] Ribas R., Ghazoui Z., Gao Q., Pancholi S., Rani A., Dunbier A. (2014). Identification of chemokine receptors as potential modulators of endocrine resistance in oestrogen receptor-positive breast cancers. Breast Cancer Res..

[43] Buluwela L., Pike J., Mazhar D., Kamalati T., Hart S.M., Al-Jehani R. (2005). Inhibiting estrogen responses in breast cancer cells using a fusion protein encoding estrogen receptor-alpha and the transcriptional repressor PLZF. Gene Ther..

[44] Tyanova S., Temu T., Sinitcyn P., Carlson A., Hein M.Y., Geiger T. (2016). The Perseus computational platform for comprehensive analysis of (prote)omics data. Nat. Methods.

[45] Oughtred R., Rust J., Chang C., Breitkreutz B.-J., Stark C., Willems A. (2021). The BioGRID database: a comprehensive biomedical resource of curated protein, genetic, and chemical interactions. Protein Sci..

[46] Mohammed H., D'Santos C., Serandour A.A., Ali H.R., Brown G.D., Atkins A. (2013). Endogenous purification reveals GREB1 as a key estrogen receptor regulatory factor. Cell Rep..

[47] Raudvere U., Kolberg L., Kuzmin I., Arak T., Adler P., Peterson H. (2019). g:Profiler: a web server for functional enrichment analysis and conversions of gene lists (2019 update). Nucleic Acids Res..

[48] Shannon P., Markiel A., Ozier O., Baliga N.S., Wang J.T., Ramage D. (2003). Cytoscape: a software environment for integrated models of biomolecular interaction networks. Genome Res..

[49] Rees J.S., Li X.-W., Perrett S., Lilley K.S., Jackson A.P. (2015). Selective proteomic proximity labeling assay using tyramide (SPPLAT): a quantitative method for the proteomic analysis of localized membrane-bound protein clusters. Curr. Protoc. Protein Sci..

[50] Kaplan P.A., Frazier S.R., Loy T.S., Diaz-Arias A.A., Bradley K., Bickel J.T. (2005). 1D5 and 6F11. Am. J. Clin. Pathol..

[51] Ambrosino C., Tarallo R., Bamundo A., Cuomo D., Franci G., Nassa G. (2010). Identification of a hormone-regulated dynamic nuclear actin network associated with estrogen receptor alpha in human breast cancer cell nuclei. Mol. Cell. Proteomics.

[52] Kim M., Park J., Bouhaddou M., Kim K., Rojc A., Modak M. (2021). A protein interaction landscape of breast cancer. Science.

[53] Shin E.M., Huynh V.T., Neja S.A., Liu C.Y., Raju A., Tan K. (2021). GREB1: an evolutionarily conserved protein with a glycosyltransferase domain links ERα glycosylation and stability to cancer. Sci. Adv..

[54] Chan C.M.W., Martin L.-A., Johnston S.R.D., Ali S., Dowsett M. (2002). Molecular changes associated with the acquisition of oestrogen hypersensitivity in MCF-7 breast cancer cells on long-term oestrogen deprivation. J. Steroid Biochem. Mol. Biol..

[55] Simigdala N., Gao Q., Pancholi S., Roberg-Larsen H., Zvelebil M., Ribas R. (2016). Cholesterol biosynthesis pathway as a novel mechanism of resistance to estrogen deprivation in estrogen receptor-positive breast cancer. Breast Cancer Res..

[56] Pancholi S., Simigdala N., Ribas R., Schuster E., Leal M.F., Nikitorowicz-Buniak J. (2022). Elacestrant demonstrates strong anti-estrogenic activity in PDX models of estrogen-receptor positive endocrine-resistant and fulvestrant-resistant breast cancer. NPJ Breast Cancer.

[57] Malorni L., Giuliano M., Migliaccio I., Wang T., Creighton C.J., Lupien M. (2016). Blockade of AP-1 potentiates endocrine therapy and overcomes resistance. Mol. Cancer Res..

[58] Oyama M., Nagashima T., Suzuki T., Kozuka-Hata H., Yumoto N., Shiraishi Y. (2011). Integrated quantitative analysis of the phosphoproteome and transcriptome in tamoxifen-resistant breast cancer. J. Biol. Chem..

[59] Shen C., Huang Y., Liu Y., Wang G., Zhao Y., Wang Z. (2011). A modulated empirical Bayes model for identifying topological and temporal estrogen receptor α regulatory networks in breast cancer. BMC Syst. Biol..

[60] Yan J., Kim Y.-S., Yang X.-P., Albers M., Koegl M., Jetten A.M. (2007). Ubiquitin-interaction motifs of RAP80 are critical in its regulation of estrogen receptor alpha. Nucleic Acids Res..

[61] Szklarczyk D., Franceschini A., Wyder S., Forslund K., Heller D., Huerta-Cepas J. (2015). STRING v10: protein-protein interaction networks, integrated over the tree of life. Nucleic Acids Res..

[62] Liu J., Wang Q., Kang Y., Xu S., Pang D. (2022). Unconventional protein post-translational modifications: the helmsmen in breast cancer. Cell Biosci..

[63] Traboulsi T., El Ezzy M., Dumeaux V., Audemard E., Mader S. (2019). Role of SUMOylation in differential ERα transcriptional repression by tamoxifen and fulvestrant in breast cancer cells. Oncogene.

[64] Liu X., Salokas K., Weldatsadik R.G., Gawriyski L., Varjosalo M. (2020). Combined proximity labeling and affinity purification-mass spectrometry workflow for mapping and visualizing protein interaction networks. Nat. Protoc..

[65] Lobingier B.T., Hüttenhain R., Eichel K., Miller K.B., Ting A.Y., von Zastrow M. (2017). An approach to spatiotemporally resolve protein interaction networks in living cells. Cell.

[66] Rhee H.-W., Zou P., Udeshi N.D., Martell J.D., Mootha V.K., Carr S.A. (2013). Proteomic mapping of mitochondria in living cells via spatially restricted enzymatic tagging. Science.

[67] Walsh C.A., Qin L., Tien J.C.-Y., Young L.S., Xu J. (2012). The function of steroid receptor coactivator-1 in normal tissues and cancer. Int. J. Biol. Sci..

[68] Belachew E.B., Sewasew D.T. (2021). Molecular mechanisms of endocrine resistance in estrogen-positive breast cancer. Front. Endocrinol. (Lausanne).

[69] Hicke L., Schubert H.L., Hill C.P. (2005). Ubiquitin-binding domains. Nat. Rev. Mol. Cell Biol..

[70] Wijayaratne A.L., McDonnell D.P. (2001). The human estrogen receptor-alpha is a ubiquitinated protein whose stability is affected differentially by agonists, antagonists, and selective estrogen receptor modulators. J. Biol. Chem..

[71] Berry N.B., Fan M., Nephew K.P. (2008). Estrogen receptor-alpha hinge-region lysines 302 and 303 regulate receptor degradation by the proteasome. Mol. Endocrinol..

[72] Jin M.L., Kim Y.W., Jin H.L., Kang H., Lee E.K., Stallcup M.R. (2018). Aberrant expression of SETD1A promotes survival and migration of estrogen receptor α-positive breast cancer cells. Int. J. Cancer.

[73] Perez-Riverol Y., Csordas A., Bai J., Bernal-Llinares M., Hewapathirana S., Kundu D.J. (2019). The PRIDE database and related tools and resources in 2019: improving support for quantification data. Nucleic Acids Res..

