# Supplementary material for: Exploring the Spatial Landscape of the Estrogen Receptor Proximal Proteome With Antibody-Based Proximity Labeling
Source: Mol Cell Proteomics. 2023 Dec 19;23(1):100702. doi: 10.1016/j.mcpro.2023.100702 (PMC10831774; doi:10.1016/j.mcpro.2023.100702)
Supplement: Supplemental Figures S1–S8 [file mmc1.docx]

Supplemental Figures

Exploring the spatial landscape of the Estrogen Receptor proximal proteome with antibody-based proximity labeling

Camilla Rega^1§^, Zuzanna Kozik^1^, Lu Yu^1^, Ifigenia Tsitsa^1^, Lesley-Ann Martin^1^, *Jyoti Choudhary*^1§^

^1^ The Institute of Cancer Research, London SW3 6JB, United Kingdom.

^§^ Corresponding author. E-mail: camilla.rega@icr.ac.uk, jyoti.choudhary@icr.ac.uk.

**List of Supplemental Figures**

**Supplementary Figure 1.** MCF7 cell lines stably express ERα-Flag in a tetracycline-inducible manner.

**Supplementary Figure 2.** Analysis of BAR ERα-Flag proximal protein candidates and comparison to known datasets.

**Supplementary Figure 3.** BAR-derived biotinylation requires H_2_O_2_, biotin phenol (BP), primary and HRP-conjugated antibodies.

**Supplementary Figure 4.** Receiver operating characteristic (ROC) analysis to validate the cut-offs used for ERα-Flag and ERα-wt BAR datasets.

**Supplementary Figure 5.** Overlap of proteins identified in our study and other ERα interactome datasets.

**Supplementary Figure 6.** Analysis of BAR ERα proximal protein candidates in cell lines modeling endocrine resistance and comparison with previously published datasets.

**Supplementary Figure 7.** ERα proximal interacting candidates and their correlation with ERα protein levels in cells modeling adaptation to endocrine resistance.

**Supplementary Figure 8.** ERα proximal protein network and its correlation with protein level changes in cells modeling adaptation to endocrine resistance.

## Supplementary Figure 1


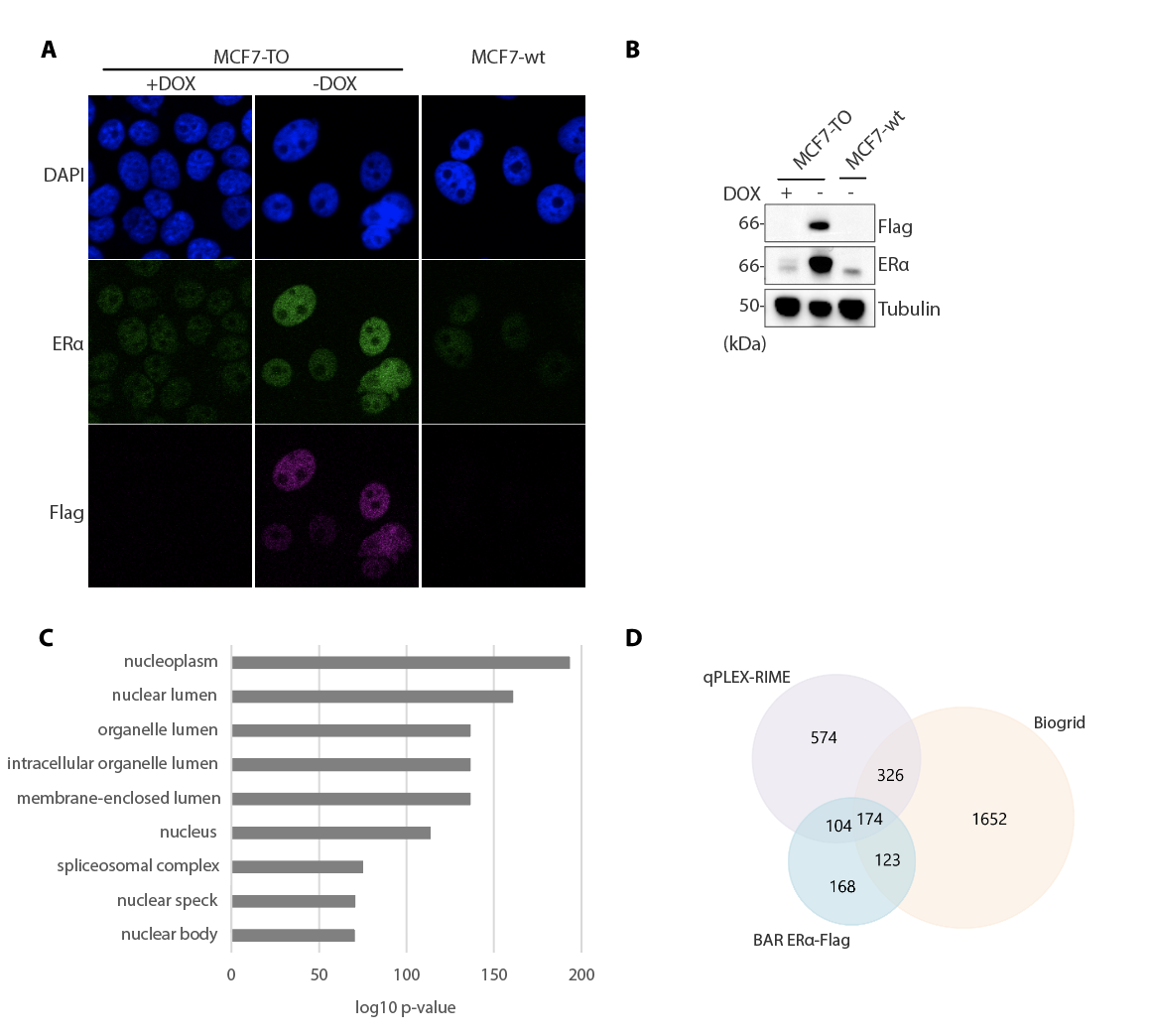


**Supplementary Figure 1. MCF7 cell lines stably express ERα-Flag in a tetracycline-inducible manner.**

(A) Confocal images of MCF7-TO cells stained with anti-Flag and anti-ERα antibodies to assess gene expression silencing in the presence of doxycycline (DOX). ERα-Flag expression was silenced upon DOX treatment. Nuclei were detected by DAPI (blue), endogenous ERα was visualized by Alexa Fluor 488 dye (green), Flag was detected by Alexa Fluor 647 dye (magenta). (B) Whole cell lysates from MCF7-TO and MCF7-wt in the presence or absence of DOX were analyzed by western blot. Tubulin was used as loading control.

## Supplementary Figure 2

##
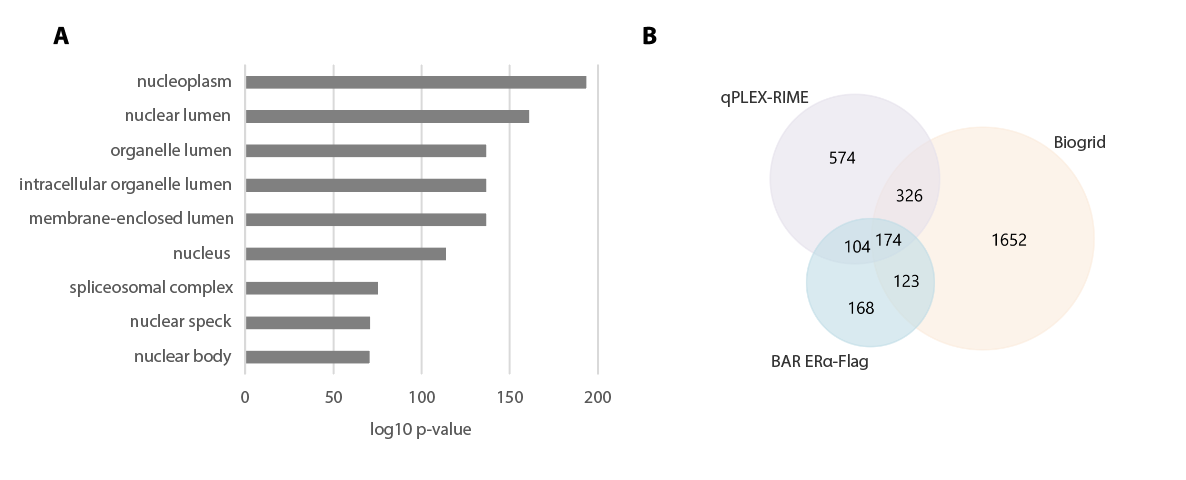


**Supplementary Figure 2. Analysis of BAR ERα-Flag proximal protein candidates and comparison to known datasets.**

(A) Gene Ontology (GO) cellular component analysis of proteins enriched in BAR ERα-Flag compared to the control. (B) Overlap of the BAR data with known ERα-associated proteins from BioGRID and qPLEX-RIME datasets.

##

## Supplementary Figure 3


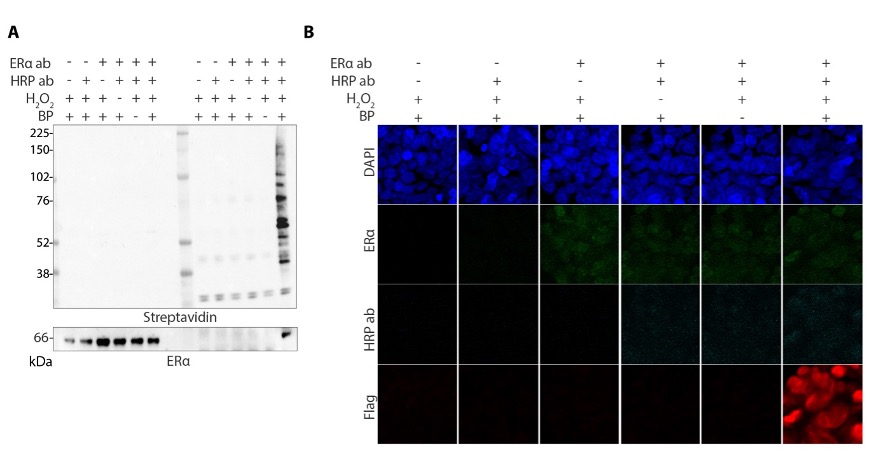


**Supplementary Figure 3. BAR-derived biotinylation requires H_2_O_2_, biotin phenol (BP), primary and HRP-conjugated antibodies.**

(A) Western blot analysis showing ERα enrichment upon streptavidin beads pull-down. Biotinylation reaction was performed omitting any of the substrates used in the proximity labeling reaction. ERα was efficiently immunoprecipitated only in the presence of all components. (B) Same analysis as in (A), by confocal microscopy. Nuclei were detected by DAPI (blue), endogenous ERα was visualized by Alexa Fluor 488 dye (green), HRP antibody was visualized by anti-goat Alexa 594 dye (cyan), biotinylated proteins were detected by streptavidin-conjugated Alexa Fluor 647 dye (red).

**Supplementary Figure 4**


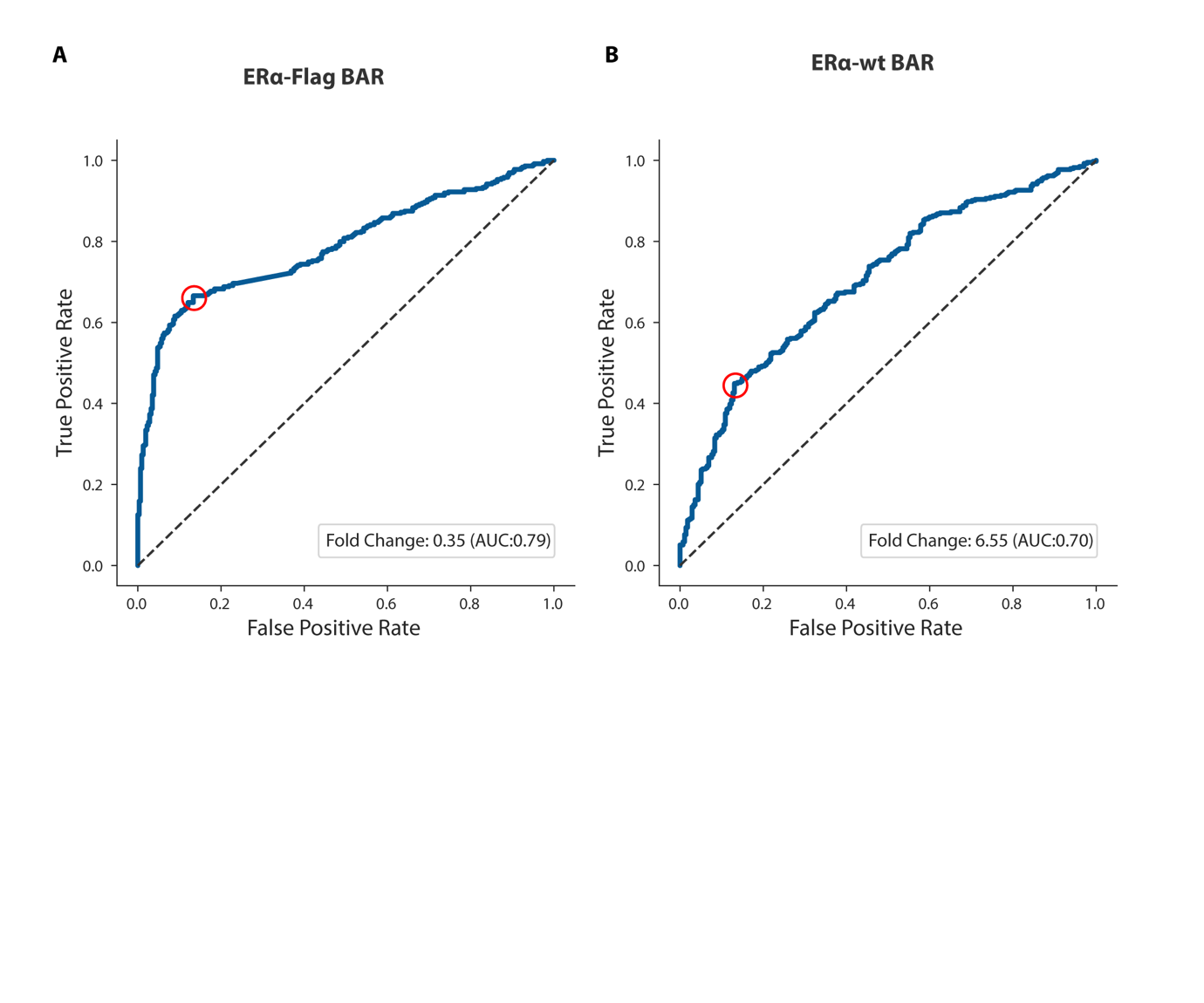


**Supplementary Figure 4. Receiver operating characteristic (ROC) analysis to validate the cut-offs used for ERα-Flag and ERα-wt BAR datasets.**

ROC curve for the (A) ERα-Flag and (B) ERα-wt BAR datasets. The curve was drawn by plotting the true positive rate against the false positive rate at fold change values. Red circle marks the selected cut-offs.

## Supplementary Figure 5

##
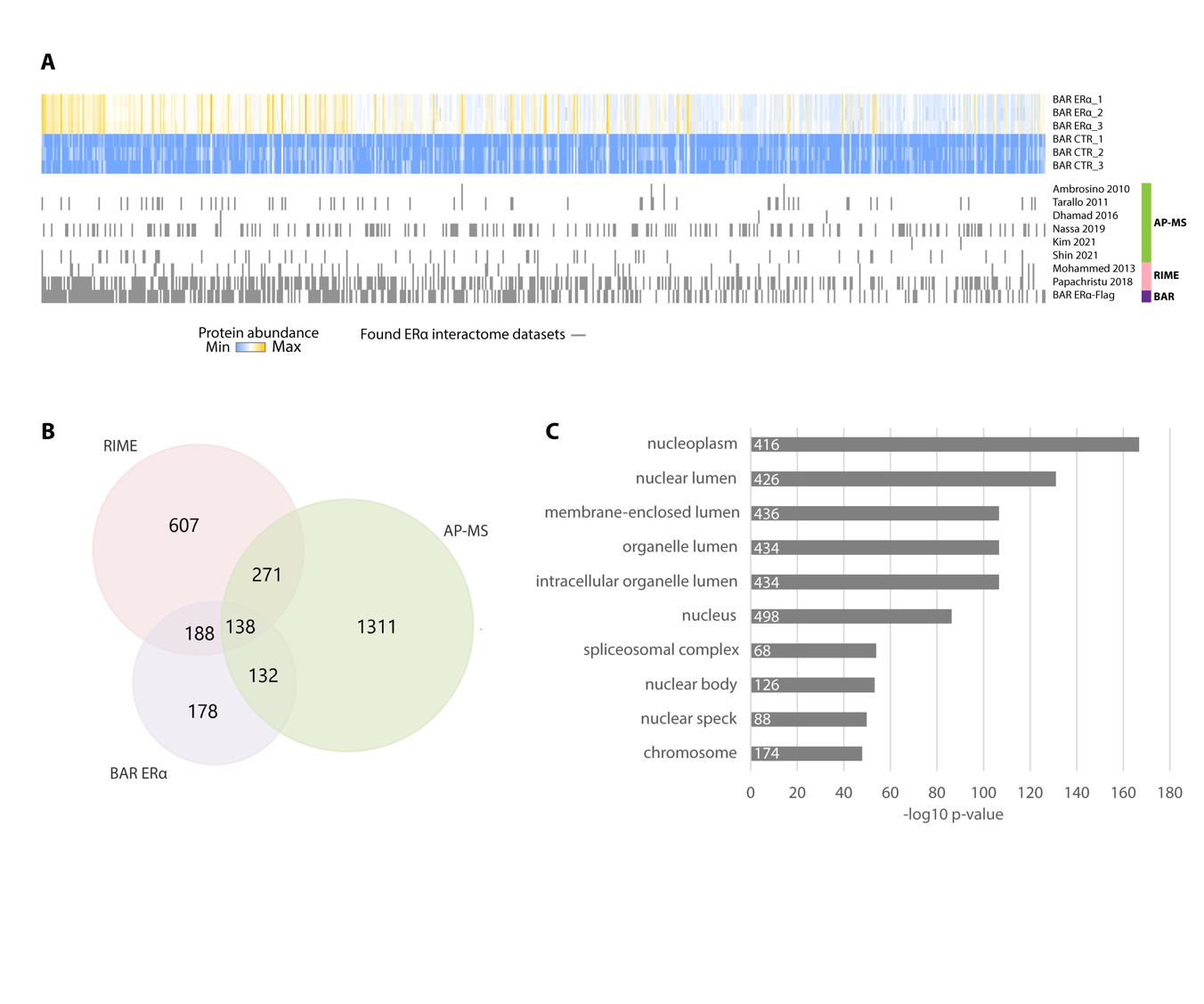


**Supplementary Figure 5. Overlap of proteins identified in our study and other ERα interactome datasets.**

(A) In the upper panel, the enrichment pattern heatmap of ERα proximal protein candidates identified in ERα BAR. Proteins are colored according to their abundance. In the lower panel, heatmap of proteins detected (gray) or not detected in other ERα interactome datasets. Datasets are grouped based on the method used in the studies (AP-MS or RIME). Proteins detected in BAR targeting ERα-Flag are also shown. (B) Venn diagram showing the overlap between published ERα interactome datasets (i.e. RIME [(28, 46)](https://sciwheel.com/work/citation?ids=1480842,5499554&pre=&pre=&suf=&suf=&sa=0,0) and AP-MS [(30, 31, 51–53)](https://sciwheel.com/work/citation?ids=5799999,12699114,14411898,11773774,14411906&pre=&pre=&pre=&pre=&pre=&suf=&suf=&suf=&suf=&suf=&sa=0,0,0,0,0) and proteins identified with BAR using the 6F11 antibody. (C) Gene Ontology (GO) cellular component analysis of ERα proximal protein candidates identified with BAR.

## Supplementary Figure 6


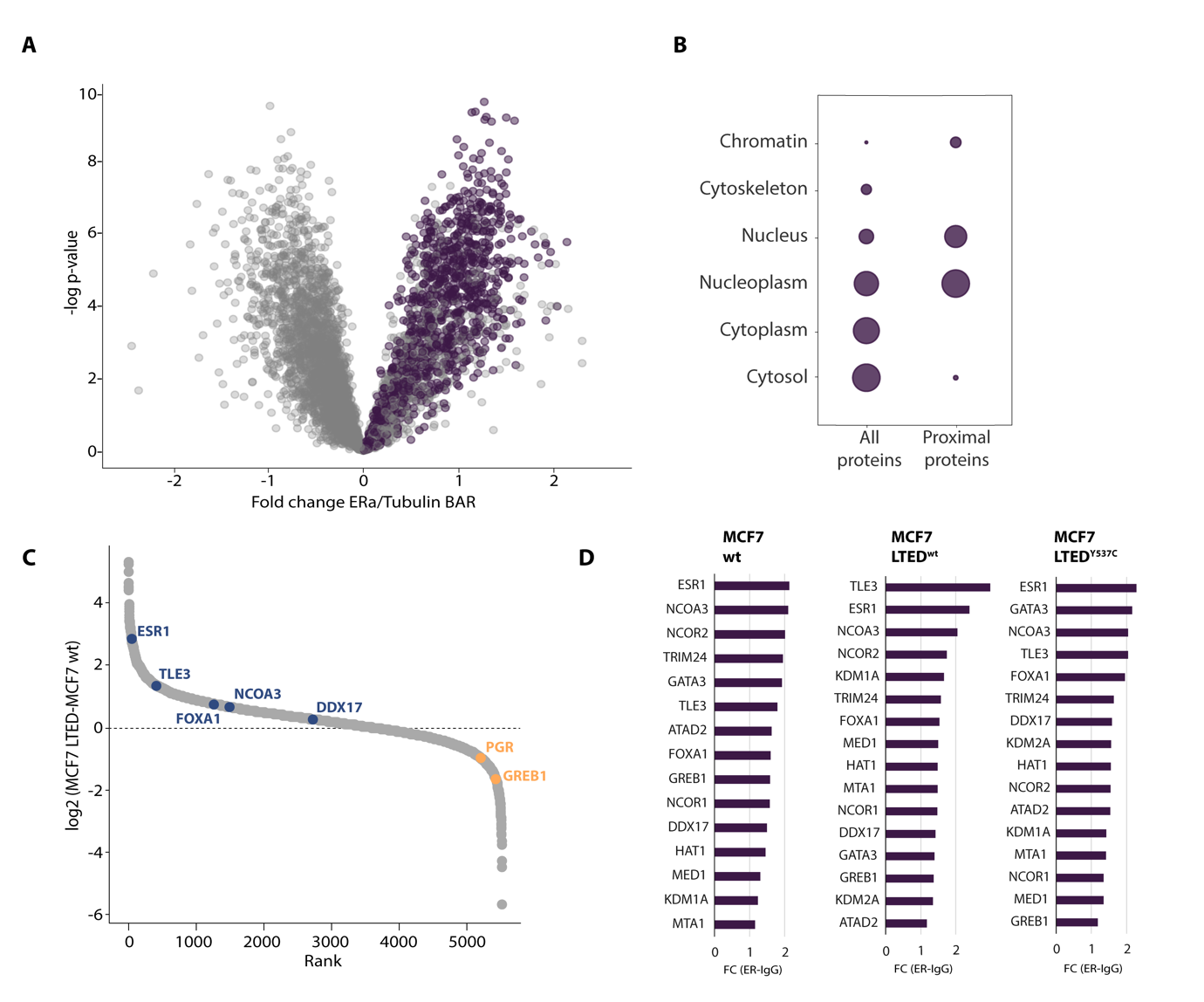


**Supplementary Figure 6. Analysis of BAR ERα proximal protein candidates in cell lines modeling endocrine resistance and comparison with previously published datasets.**

(A) Volcano plot showing proteins enriched in biotinylated *versus* control samples in the BAR on MCF7-wt, MCF7-LTED^wt^ and MCF7-LTED^Y537C^ analysis. Refined ERα proximal protein candidates exhibiting greater protein abundance in BAR ERα compared to the spatial reference and negative control (tubulin BAR) are shown in purple. Proteins non statistically significant (student t-test q-value> 0.01) are shown in gray. (B) Gene Ontology (GO) cellular component analysis before and after filtering out non-proximal proteins using spatial control (tubulin BAR). Node size represents adjusted p-values. (C) Rank plot showing differentially abundant proteins in MCF7-wt and the MCF7-LTED^wt^ from our previously published dimethyl labeling proteomics data [(55)](https://sciwheel.com/work/citation?ids=3705057&pre=&suf=&sa=0). Protein names referred to in “Results'' are labeled. (D) Bar plots summarizing the ERα  interaction protein changes in MCF7-wt, MCF7-LTED^wt^ and MCF7-LTED^Y537C^ cell lines identified in our previously published qPLEX-RIME dataset [(56)](https://sciwheel.com/work/citation?ids=14414087&pre=&suf=&sa=0). Proteins are ranked by log_2_ fold change difference (ERα-IgG) RIME.

## Supplementary Figure 7


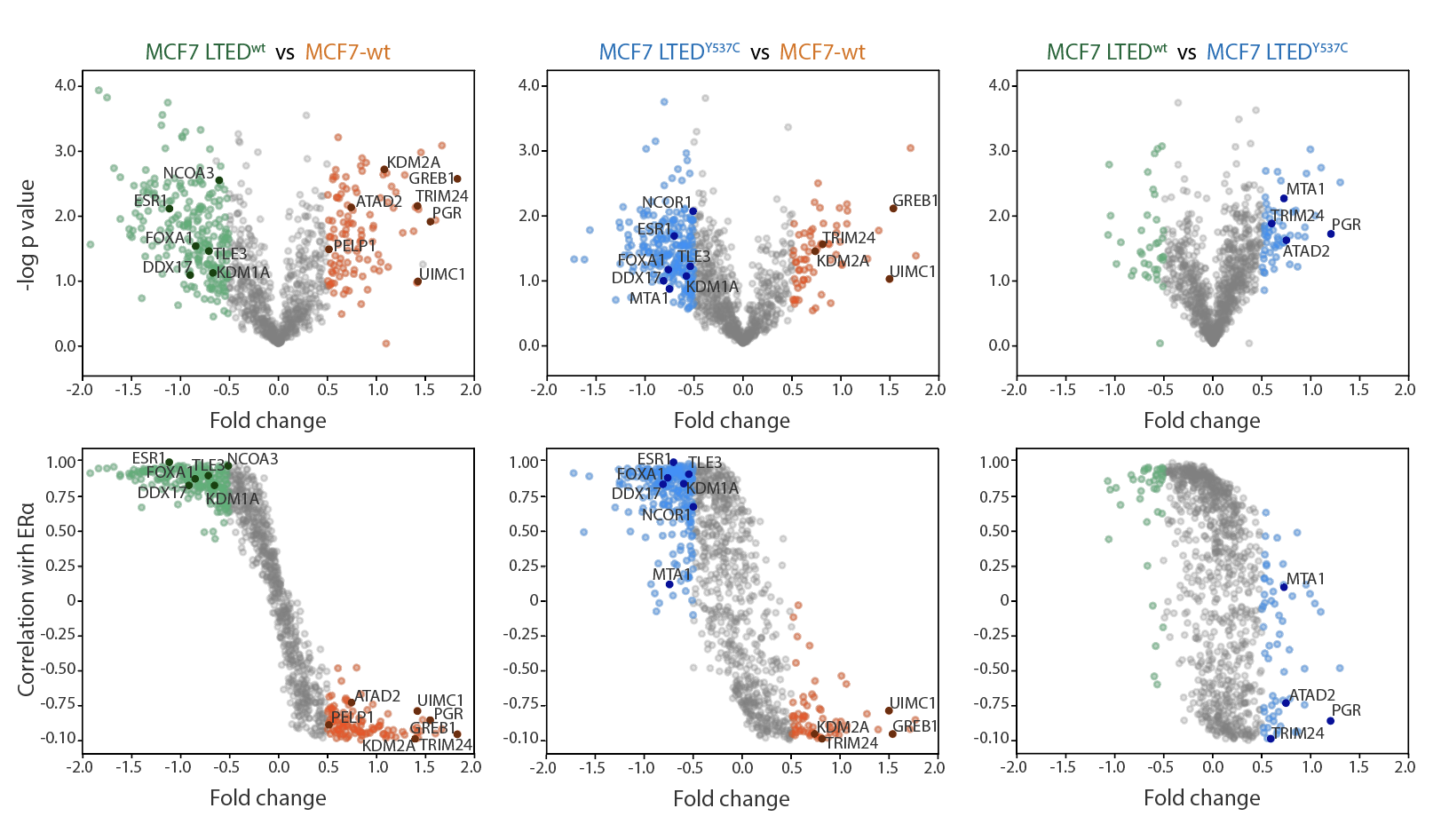


**Supplementary Figure 7. ERα proximal protein candidates and their correlation with ERα protein levels in cells modeling adaptation to endocrine resistance.**

ERα proximal protein candidates significantly enriched in each MCF7 cell line (Student’s t-test, q-value <0.05, S0=2) and their correlation with ERα expression levels. Volcano plots on the top show proteins significantly enriched in MCF7-LTED^wt^, MCF7-wt and MCF7-LTED^Y537C^ highlighted in green, orange and turquoise, respectively. Scatter plots in the bottom show their correlation with ERα expression (Pearson’s correlation). Known ERα interactors are highlighted.

## Supplementary Figure 8


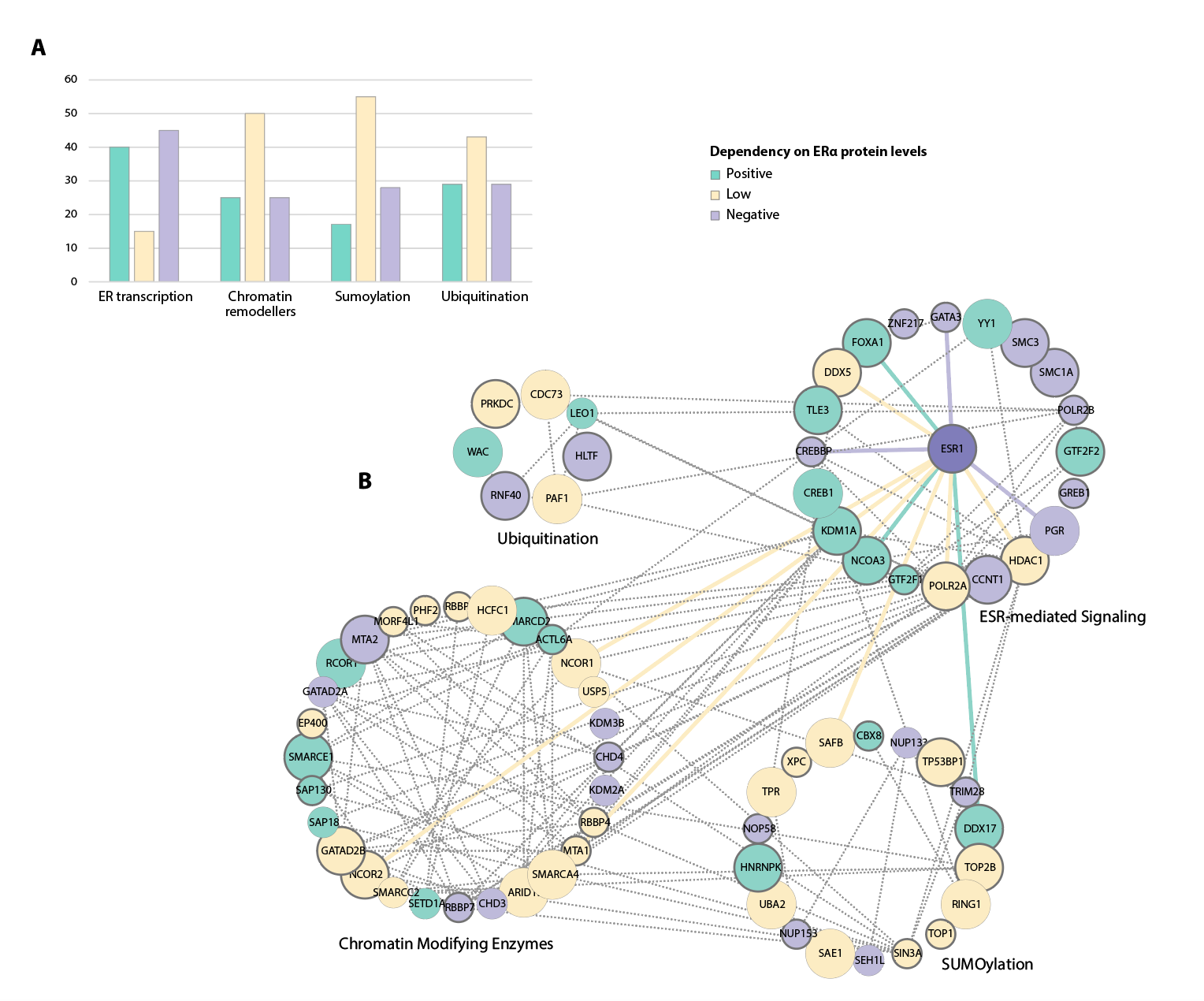


**Supplementary Figure 8. ERα proximal protein network and its correlation with protein level changes in cells modeling adaptation to endocrine resistance.**

(A) Bar plot showing the percentage of proteins within the ERα proximal proteome classified into clusters based on their correlation with ERα levels in cells modeling adaptation to acquired resistance, further grouped based on biological function. (B) Network of the ERα proximity proteins organized by relevant biological processes. Individual proteins are shown as nodes and interactions as edges. Proteins are colored according to ERα expression clusters. Bigger nodes indicate protein identified in three out of three independent BAR datasets. ERα first neighbor interactions are shown with thicker lines and colored according to cluster.
